# NELL-1 in the treatment of osteoporotic bone loss

**DOI:** 10.1038/ncomms8362

**Published:** 2015-06-17

**Authors:** Aaron W. James, Jia Shen, Xinli Zhang, Greg Asatrian, Raghav Goyal, Jin H. Kwak, Lin Jiang, Benjamin Bengs, Cymbeline T. Culiat, A. Simon Turner, Howard B. Seim III, Benjamin M. Wu, Karen Lyons, John S. Adams, Kang Ting, Chia Soo

**Affiliations:** 1 Department of Orthopaedic Surgery and the Orthopaedic Hospital Research Center, UCLA and Orthopaedic Hospital, University of California, Los Angeles, California 90095, USA; 2 Division of Growth and Development, Section of Orthodontics, School of Dentistry, University of California, Los Angeles, California 90095, USA; 3 Department of Pathology and Laboratory Medicine, David Geffen School of Medicine, University of California, Los Angeles, California 90095, USA; 4 Department of Neurology, Easton Center for Alzheimer's Disease Research, Molecular Biology Institute, University of California, Los Angeles, California 90095, USA; 5 Oak Ridge National Laboratory (ORNL), Oak Ridge, Tennessee 37830, USA; 6 Department of Veterinary Sciences, Colorado State University, Fort Collins, Colorado 80523, USA; 7 Department of Bioengineering and Department of Material Sciences, University of California, Los Angeles, California 90095, USA; 8 Division of Plastic and Reconstructive Surgery, Department of Surgery, David Geffen School of Medicine, University of California, Los Angeles, Los Angeles, California 90095, USA

## Abstract

NELL-1 is a secreted, osteoinductive protein whose expression rheostatically controls skeletal ossification. Overexpression of NELL-1 results in craniosynostosis in humans and mice, whereas lack of *Nell-1* expression is associated with skeletal undermineralization. Here we show that *Nell-1*-haploinsufficient mice have normal skeletal development but undergo age-related osteoporosis, characterized by a reduction in osteoblast:osteoclast (OB:OC) ratio and increased bone fragility. Recombinant NELL-1 binds to integrin β1 and consequently induces Wnt/β-catenin signalling, associated with increased OB differentiation and inhibition of OC-directed bone resorption. Systemic delivery of NELL-1 to mice with gonadectomy-induced osteoporosis results in improved bone mineral density. When extended to a large animal model, local delivery of NELL-1 to osteoporotic sheep spine leads to significant increase in bone formation. Altogether, these findings suggest that NELL-1 deficiency plays a role in osteoporosis and demonstrate the potential utility of NELL-1 as a combination anabolic/antiosteoclastic therapeutic for bone loss.

## Abstract (web summary)

The growth factor NELL-1 induces bone formation during development, but its role in osteoporosis is unknown. This study shows that NELL-1 binding to integrin ß1 induces Wnt/ß-catenin signalling in the bone and restores bone mineral density in osteoporotic mice and sheep, suggesting the therapeutic potential of NELL-1 for the treatment of bone loss.

## Graphical abstract

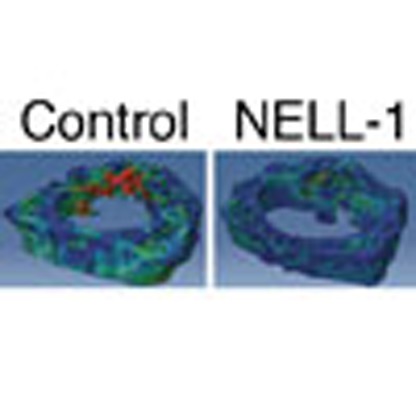

Osteoporosis is a disease of severe bone loss affecting an estimated 10 million Americans and causing two million pathological fractures per year^1^. Osteoporosis results from an imbalance between bone formation and resorption. This balance depends on both the number of osteoblastic (OB) and osteoclastic (OC) cells as well as their cellular activity within the bone metabolic unit^2^,^3^,^4^. Typically, bone anabolic agents produce a secondary osteoclastogenic response, as is the case for PTH, BMP2 and retinoic acid^5^,^6^,^7^,^8^,^9^. The interest in therapies with the potential to uncouple OB and OC activity has led to intense focus on activation of the Wnt/β-catenin signalling pathway, as this pathway has predominant anabolic effects on bone. However, high lipophilicity and insolubility make recombinant Wnt proteins challenging for bioactive delivery^10^,^11^,^12^. An alternative approach is to increase levels of Wnt signalling via inactivation of endogenous Wnt inhibitors (Sclerostin (SOST) and DKK-1). In fact, treatment with anti-SOST and anti-DKK-1 antibodies results in increased OB number and activity, reduced OC number and activity, and a consequent increase in bone mineral density (BMD)^13^,^14^, observed in rat^15^,^16^, non-human primate^17^ and human studies^18^. In aggregate, positive regulation of the Wnt/β-catenin signalling pathway has emerged as a promising new field for anabolic, anti-OC therapies in osteoporosis.

NELL-1 is a unique secreted protein of 810 amino acids first studied in the context of human craniofacial skeletal development, where NELL-1 was noted to be osteoinductive and its overexpression associated with human craniosynostosis (CS)^19^. Since that time, transgenic *Nell-1*-overexpressing mice have been observed to recapitulate a CS-like phenotype^20^. Conversely, *Nell-1*-deficient mice (as developed by N-ethyl-N-nitrosourea (ENU)-induced mutagenesis) exhibit cranial and vertebral bone defects with undermineralization^21^. Mechanistically, NELL-1 binds to the cell surface receptor integrin β1 (ref. 22) and regulates activity of the master osteogenic transcription factor, Runt-related transcription factor-2 (Runx2)^23^. Despite the well-demonstrated osteogenic effects of NELL-1 (refs 24, 25, 26, 27), there has been little understanding of the signalling pathway through which the effects of NELL-1 are exerted, nor the potential role of NELL-1 in osteoporosis. However, a recent genome-wide study of single-nucleotide polymorphisms found a linkage between *NELL-1* and osteoporosis in human patients^28^.

Here we explore the causative and therapeutic possibilities of manipulating NELL-1 signalling in osteoporosis. First, the effects of *Nell-1* deficiency in skeletal aging were evaluated by studying the skeletons of *Nell-1*-haploinsufficient mice. Next, the opposing effects of NELL-1 on OB and OC cells as well as mechanisms underlying NELL-1's activation of Wnt/β-catenin signalling were uncovered. Further, a large animal study was performed in which surgical delivery of NELL-1 was used in order to combat osteoporotic bone loss in sheep spine. Finally, systemic administration of NELL-1 was used to reverse gonadectomy-induced osteoporotic bone loss in mice. In sum, the present study describes a new role for NELL-1 as an aetiological factor and a treatment of osteoporosis.

## Results

### Osteoporotic phenotype of Nell-1-haploinsufficient mouse

**Figure 1:**
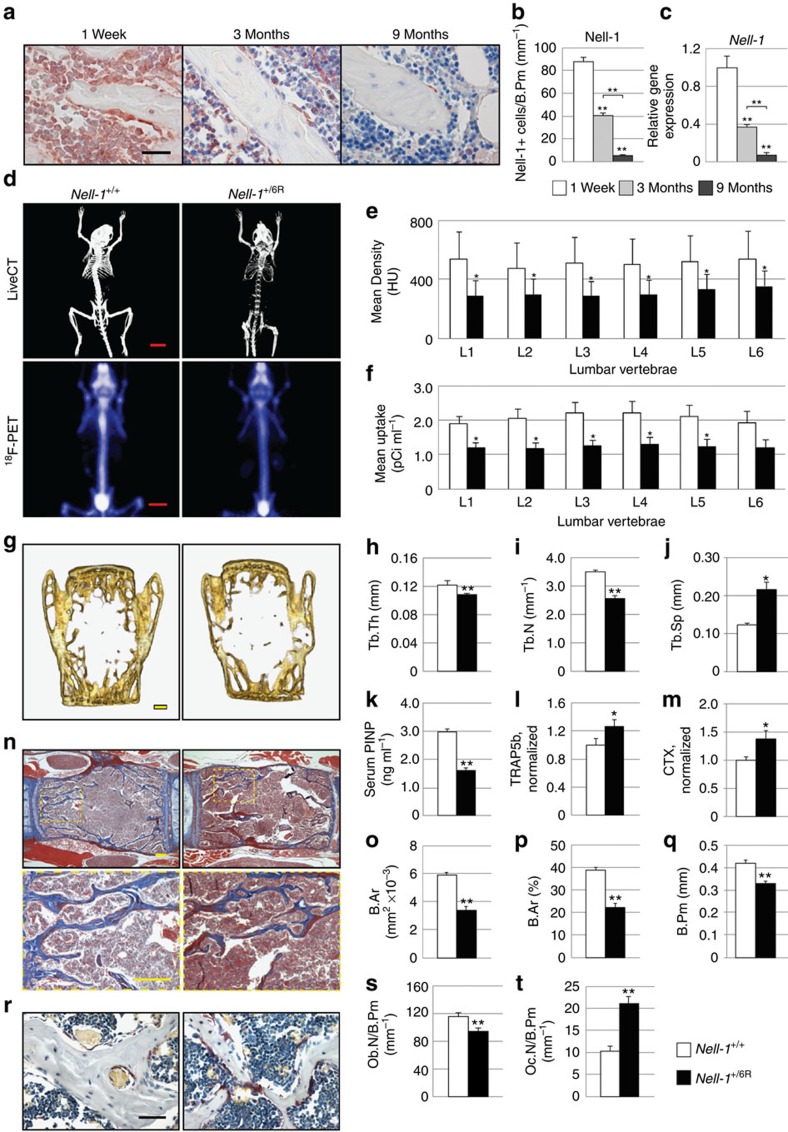
Osteoporotic phenotype of aged *Nell-1*-haploinsufficient mice. (**a**) Immunohistochemical staining for Nell-1 protein in rat spines, at 1 week, and 3 and 9 months. (**b**) Semiquantification of Nell-1 immunohistochemical staining, expressed as Nell-1^+^ bone-lining cells per B.Pm (*N*=20, 39 and 25 images, respectively). (**c**) Relative *Nell-1* gene expression in mouse spines at 1 week, and 3 and 9 months, using qRT–PCR (*N*=3 samples per time point). (**d**) 3D image of liveCT and ^18^F radioisotope incorporation of aged (18-month old) wild-type (*Nell-1*^+/+^) and *Nell-1*-haploinsufficient (*Nell-1*^+/6R^) mice. (**e**) Quantification of BMD stratified by the lumbar vertebral level (L1–L6; *N*=11 and 12 mice, respectively). (**f**) Quantification of ^18^F incorporation (*N*=6 mice per genotype). (**g**) 3D coronal reconstructions of lumbar vertebrae of aged *Nell-1*^+/+^ and *Nell-1*^+/6R^ mice. (**h–j**) Trabecular analyses of the lumbar vertebrae, including (**h**) trabecular bone thickness (Tb.Th), (**i**) number (Tb.N) and (**j**) spacing (Tb.Sp; *N*=12 and 19 vertebrae, respectively). (**k–m**) Serum studies among *Nell-1*^+/+^ and *Nell-1*^+/6R^ aged mice including (**k**) serum PINP (*N*=19 and 17 mice), (**l**) serum TRAP5b (*N*=10 and 9 mice) and (**m**) serum CTX (*N*=12 and 22 mice). (**n**) Masson's trichrome staining of lumbar vertebral bodies of *Nell-1*^+/+^ and *Nell-1*^+/6R^ mice, coronal section. (**o–q**) Histomorphometric quantifications of lumbar vertebral bodies. Measurements include (**o**) B.Ar (*N*=29 and 26 images), (**p**) percentage (%) B.Ar (*N*=29 and 26 images) and (**q**) B.Pm (*N*=30 and 29 images). (**r**) TRAP staining of lumbar vertebral bodies, indicating osteoclasts. Quantification of (**s**) Ob.N per B.Pm (*N*=20 images per genotype) and (**t**) Oc.N per B.Pm (*N*=26 and 39 images). Black scale bar, 25 μm; red scale bar, 10 mm; yellow scale bar, 100 μm. HU: Hounsfield Units; pCi (pico Curie). Data points indicate the means, while error bars represent one s.e.m. Experiments were performed without replicate. Parametric data were analysed using an appropriate Student's *t*-test or a one-way analysis of variance (ANOVA), followed by a *post hoc* Tukey's test. Nonparametric data were analysed with a Mann–Whitney *U*-test or a Kruskal–Wallis one-way analysis. *P<0.05, **P<0.01.

NELL-1 expression, known to be present during skeletal development, was evaluated with skeletal aging. Nell-1 protein showed a significant reduction with age, as shown by immunohistochemistry of trabecular bone-lining OBs in the rat lumbar spine (Fig. 1a). Semiquantitative analysis demonstrated a significant decrease in Nell-1^+^ bone-lining cells with age (Fig. 1b). These results were further quantified using quantitative reverse transcription (qRT)–PCR, showing a progressive loss of *Nell-1* in the bone with age (Fig. 1c). The consequences of *Nell-1* deficiency on skeletal aging were next assessed. Deficiency was induced by the generation of a point mutation in the *Nell-1* gene (*Nell-1*^6R/6R^) resulting in a premature stop codon and near complete loss of transcript levels. Complete deficiency (*Nell-1*^6R/6R^) is neonatal lethal with major skeletal anomalies, while the heterozygote (*Nell-1*^+/6R^) mice are without gross abnormalities at birth^21^. The *Nell-1*^+/6R^ mouse skeleton was studied across its lifetime. The neonatal (P1 days), young adult (1 month) and adult skeleton (6 months) showed no significant skeletal phenotype, as assessed by combined live CT/fluoride radioisotope (^18^F) incorporation studies, and histomorphometric analyses (Supplementary Fig. 1, Supplementary Table 1). In contrast, by 18 months of life the *Nell-1*^+/6R^ mouse developed significant osteoporosis (Fig. 1, Supplementary Fig. 2). This was apparent both in analysis of the lumbar spine (Fig. 1d–t) and the distal femur (Supplementary Fig. 2f–k). Analyses showed significantly reduced BMD and reduced ^18^F incorporation (Fig. 1d–f). Dual energy X-ray absorptiometry (DXA), high-resolution microCT and trabecular bone analyses confirmed an osteoporotic phenotype in the aged *Nell-1*^+/6R^ mouse in both the lumbar spine and femur (Fig. 1g–j, Supplementary Fig. 2a–k). Further, the aged *Nell-1*^+/6R^ bone showed increased bone fragility and decreased bone stiffness as shown by both computer-simulated biomechanical compression testing as well as microindentation testing (Supplementary Fig. 2l–r). Next, serum biomarkers evaluated total OB and OC activity (Fig. 1k–m). Total bone formation activity (serum Procollagen I N-terminal Propeptide (PINP)) was reduced among *Nell-1*^+/6R^ mice, while total bone resorption activity was increased in *Nell-1*^+/6R^ mice (serum Tartrate Resistant Acid Phosphatase 5b (TRAP5b) and C-Terminal Telopeptide (CTX)). Histological analysis confirmed a significant reduction in the vertebral body trabecular bone among *Nell-1*^+/6R^ specimens (Fig. 1n–q, Supplementary Fig. 2s–y). Next, OB and OC prevalence was analysed histologically (Fig. 1r–t). OB number per bone perimeter (Ob.N per B.Pm) was significantly reduced in the *Nell-1*^+/6R^ lumbar vertebrae. Next, OC numbers, evaluated by TRAP staining, showed a significant increase in osteoclast number (Oc.N) per B.Pm among *Nell-1*^+/6R^ vertebrae. Thus, by radiographic, biomechanical and histologic analyses, *Nell-1* haploinsufficiency results in normal skeletogenesis and development, but results in an osteoporotic defect with age. This osteoporotic defect, manifested in both sexes, is characterized by increased cortical bone fragility, altered trabecular bone morphology, decreased OB and increased OC number.

### NELL-1 has inverse effects on bone formation and resorption

**Figure 2:**
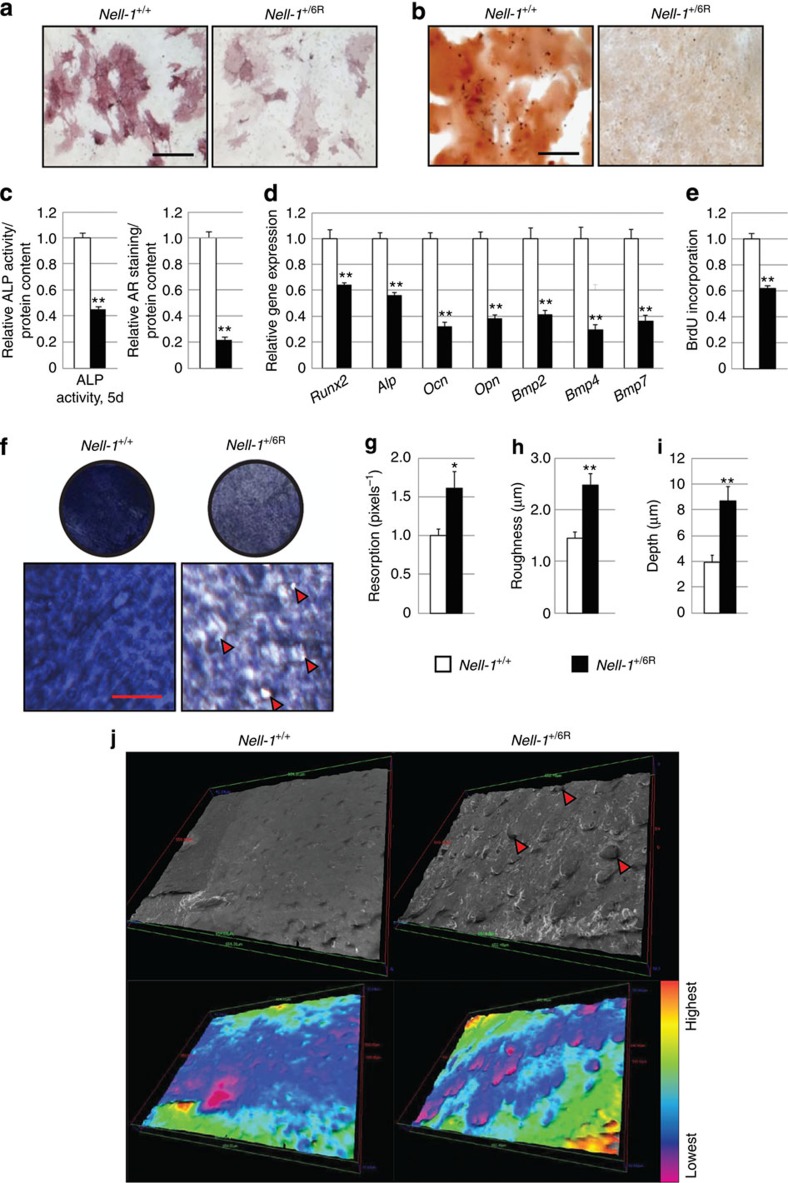
*Nell-1*^+/6R^ mice exhibit impaired osteoblastic and excessive osteoclastic activity. OB or OC precursors were derived from the bone marrow of aged (18-month old) wild-type (*Nell-1*^+/+^) and *Nell-1*-haploinsufficient (*Nell-1*^+/6R^) mice. (**a–d**) *In vitro* osteogenic differentiation assays of OB precursors from *Nell-1*^+/+^ and *Nell-1*^+/6R^ mice. (**a**) ALP staining, at 5-day osteogenic differentiation. (**b**) Alizarin red (AR) staining of bone nodules, at 10-day osteogenic differentiation. (**c**) Photometric quantification of ALP and AR activity, normalized to total protein content (*N*=3 mice and nine wells per genotype). (**d**) Relative gene expression among undifferentiated *Nell-1*^+/+^ and *Nell-1*^+/6R^ OB precursors using qRT–PCR including *Runx2*, *Alp*, *Osteocalcin* (*Ocn*), *Osteopontin* (*Opn*), *Bmp (Bone Morphogenetic Protein) 2, 4* and *7* (*N*=3 mice and three wells per genotype). (**e**) Bromodeoxyuridine (BrdU) incorporation assays of OB precursors after 72-h growth. (**f–j**) *In vitro* bone resorption assays of OC precursors derived from aged *Nell-1*^+/+^ and *Nell-1*^+/6R^ mice, performed in the presence of M-CSF and soluble RANKL (*N*=3 mice and six wells per genotype). (**f**) Calvarial disc bone resorption assays with OC precursors from *Nell-1*^+/+^ and *Nell-1*^+/6R^ mice, as shown by Toluidine blue staining after 5-day resorption. Red arrowheads indicate resorption pits. (**g**) Photographic quantification of bone resorption, as determined by Toluidine blue staining (*N*=3 mice per genotype, and 9 and 11 resorption discs, respectively). (**h,i**) Quantification of calvarial resorption as assessed by s.e.m. of (**h**) the mean bone surface roughness (*N*=36 and 29 measurements) and (**i**) the mean resorption pit depth (*N*=9 measurements per genotype). (**j**) Reconstructions of representative resorption assays on the basis of s.e.m. Red arrows indicate representative resorption pits. Colourized s.e.m. highlights resorption pits in purple. Converse experiments in which recombinant NELL-1 is added are presented in Supplementary Fig. 3. Black scale bar, 55 μm; red scale bars, 20 μm. Data points indicate the means, while error bars represent one s.e.m. *In vitro* experiments were performed in biological triplicate, unless otherwise described. Parametric data were analysed using an appropriate Student's *t*-test or a one-way ANOVA, followed by a *post hoc* Tukey's test. Nonparametric data were analysed with a Mann–Whitney *U*-test or a Kruskal–Wallis one-way analysis. **P<0.01.

*In vivo* studies demonstrated marked alterations in both OB/OC number and activity. Next, the isolated cellular effects of *Nell-1* haploinsufficiency were determined by the culture of either OB or OC precursors. First, marrow-derived *Nell-1*^+/6R^ OB precursors were examined (Fig. 2a–e). Consistent with *in vivo* findings, *Nell-1*^+/6R^ OB precursors showed a significant reduction in expression across all markers of *in vitro* osteogenic differentiation, including alkaline phosphatase (ALP)-positive cells, as well as alizarin red-positive bone nodules. Specific gene expression showed a global reduction across both ‘early' and ‘later' markers of OB differentiation. In addition, OB precursor proliferation was reduced among *Nell-1*^+/6R^ cells. Next, converse experiments were performed with OC precursors (Fig. 2f–j). OC precursors from *Nell-1*^+/+^ and *Nell-1*^+/6R^ aged littermates were seeded on bone discs for resorption assays. By gross photographic and scanning electron microscopy assessments, greater bone resorption was detected among *Nell-1*^+/6R^ OC precursors. These results were quantified, showing an increase in resorption by *Nell-1*^+/6R^ OC precursors (including topographic roughness and resorption pit depth). In summary, *Nell-1*^+/6R^ OB precursors showed a reduction in proliferation, differentiation and bone nodule formation. Conversely, *Nell-1*^+/6R^ OC precursors showed increased OC activity and bone resorption when compared with *Nell-1*^+/+^ cells.

Having demonstrated the effect of *Nell-1* deficiency in OB and OC precursors, we next enquired as to the effects of NELL-1 gain-of-function via the addition of NELL-1 protein. To answer this, marrow-derived wild-type OB and OC precursors were harvested, and identical assays *in vitro* performed, now in the presence of recombinant human (rh)NELL-1 (Supplementary Fig. 3). As expected, rhNELL-1 dose-dependently increased OB precursor osteogenic differentiation by all markers. In contrast, culture of OC precursors with rhNELL-1 led to reduced bone resorption. Thus, rhNELL-1 had contrary effects on OB and OC precursors: rhNELL-1 increased OB precursor differentiation but inhibited OC precursor differentiation/bone resorption.

### NELL-1 increases Wnt/β-catenin signalling via integrin β1

**Figure 3:**
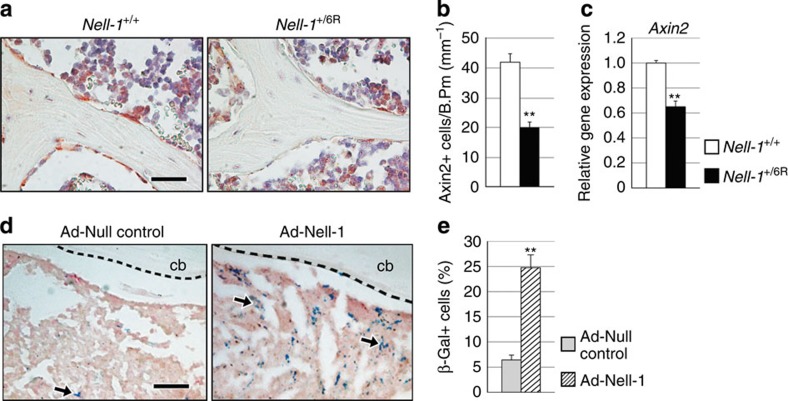
Nell-1 signalling activates Wnt/β-catenin signalling activity *in vivo*. (**a–d**) Evaluation of Wnt/β-catenin signalling in *Nell-1*^+/6R^ animals. (**a**) Axin2 immunohistochemistry in aged (18-month old) lumbar spine specimens of *Nell-1*^+/+^ and *Nell-1*^+/6R^ animals, appearing brown. Haemotoxylin counterstain appears purple. (**b**) Semiquantification of Axin2 immunohistochemical staining among trabecular bone-lining cells (*N*=32 and 22 images). (**c**) *Axin2* mRNA expression in bone marrow flush of aged (18-month old) *Nell-1*^+/+^ and *Nell-1*^+/6R^ mice, evaluated using qRT–PCR (*N*=4 samples per genotype). (**d,e**) Evaluation of Wnt/β-catenin signalling in TOPGAL reporter mice in the context of *Nell-1* overexpression. Wnt/β-catenin signalling activity was assessed after intrafemoral injection of either *Nell-1*-expressing adenovirus (Ad-Nell-1 or Ad-Null control, 5 × 10^12 ^pt ml^−1^). (**d**) Xgal staining in TOPgal femurs at 1 week post injection. Blue staining indicates Wnt-responsive cells, indicated by black arrows. cb: cortical bone (*N*=3 mice per treatment group). (**e**) FACS analysis of femoral TOPgal bone marrow flush for β-gal positivity, indicating Wnt-responsive cells. Percentage of β-gal+ cells expressed as a portion of CD45− marrow cells (non-haematopoietic cells); analysis performed 2 weeks post Ad-Nell-1 injection (*N*=4 mice per treatment group). Black scale bar, 25 μm. Data points indicate the means, while error bars represent one s.e.m. *In vivo* experiments were performed without replicate, unless otherwise described. Parametric data were analysed using an appropriate Student's *t*-test or a one-way ANOVA, followed by a *post hoc* Tukey's test. Nonparametric data were analysed with a Mann–Whitney *U*-test or a Kruskal–Wallis one-way analysis. **P<0.01.

The divergent effects of NELL-1 on OB and OC cells parallel the known effects of Wnt/β-catenin signalling^29^. To assess this link, Wnt signalling activation in the aged *Nell-1*^+/6R^ spine was compared with *Nell-1*^+/+^ littermates (Fig. 3a–c). Axin2 immunohistochemistry was first examined as a marker of Wnt signalling activation. A significant reduction in Axin2 expression was observed among bone-lining OBs as well as in marrow cells in the *Nell-1*^+/6R^ spine (Fig. 3a), confirmed using semiquantitative analysis of bone-lining cells (Fig. 3b). This reduction was further quantified using qRT–PCR, to reveal an ∼35% reduction in *Axin2* expression (Fig. 3c). Next, gain-of-function experiments were performed, using a TOPGAL Wnt reporter mouse (Fig. 3d,e). Here adenoviral *Nell-1* (Ad-Nell-1)^30^ was injected into the femoral bone marrow cavity. In comparison with control virus, Ad-Nell-1 led to a significant increase in Wnt/β-catenin signalling activity as shown by the number of β-gal^+^ marrow cells (Fig. 3d), confirmed using flow cytometry (Fig. 3e). Thus, NELL-1 loss- or gain-of-function led to reduced or increased intramarrow Wnt/β-catenin signalling, respectively.

**Figure 4:**
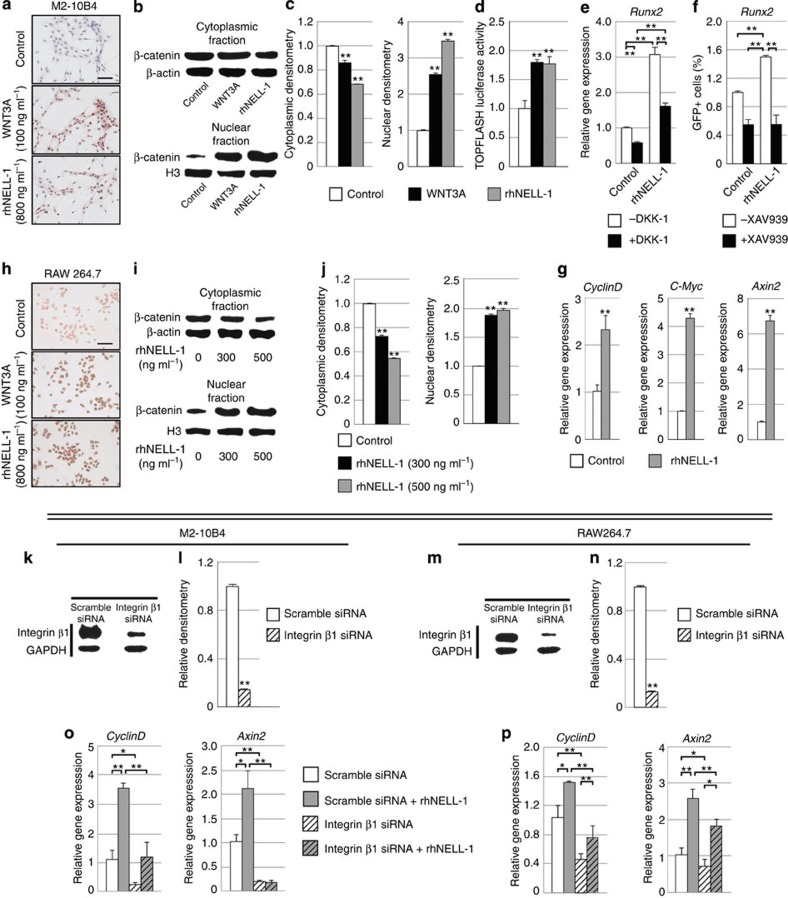
NELL-1 requires integrin β1 to activate Wnt/β-catenin signalling. (**a–d**) RhNELL-1 increases Wnt signalling in the M2-10B4 BMSC line. (**a**) Active β-catenin immunocytochemistry in M2-10B4 cells, treated with rhNELL-1, WNT3A or control (PBS). (**b,c**) Western blot analysis and quantification of cytoplasmic and nuclear β-catenin (*N*=3 wells per treatment). (**d**) M2-10B4 cells were transfected with TOPFLASH reporter (*N*=4 wells per treatment). (**e,f**) RhNELL-1 requires intact Wnt/β-catenin signalling for induction of OB differentiation (*N*=4 wells per treatment). (**e**) M2-10B4 cells were treated with PBS or rhNELL-1 with or without DKK-1 for 3 days. *Runx2* expression measured using qRT–PCR. (**f**) M2-10B4 cells were transduced with *Runx2*-EGFP reporter lentivirus and treated with PBS or rhNELL-1 with or without XAV939. *Runx2* reporter assay was performed after 3 days. (**g–i**) RhNELL-1 increases Wnt signalling in the RAW264.7 osteoclast cell line (*N*=3 wells per treatment). (**g**) Gene expression after 2 days with or without rhNELL-1. (**h**) Active β-catenin immunocytochemistry in RAW264.7 cells, treated with rhNELL-1, WNT3A or control (PBS). (**i,j**) Western blot and quantification with or without rhNELL-1. (**k–p**) RhNELL-1 requires integrin β1 to activate Wnt/β-catenin signalling (*N*=3 wells per treatment). (**k,l**) siRNA-mediated knockdown of the known NELL-1 receptor integrin β1 was performed in M2-10B4 cells, confirmed by western blot analysis and quantification. (**m,n**) Similar siRNA-mediated knockdown of integrin β1 was performed in RAW264.7 OC cells. (**o,p**) After 2 days of rhNELL-1 (300 ng ml^−1^) treatment, Wnt signalling gene expression was evaluated in either scramble or integrin β1 siRNA-treated M2-10B4 or RAW264.7 cells. Quantitative RT–PCR for *CyclinD* and *Axin2* was performed. Black scale bars, 100 μm. Data points indicate the means, while error bars represent one s.e.m. *In vitro* experiments were performed in biological triplicate, unless otherwise described. Parametric data were analysed using an appropriate Student's *t*-test or a one-way ANOVA, followed by a *post hoc* Tukey's test. Nonparametric data were analysed with a Mann–Whitney *U*-test or a Kruskal–Wallis one-way analysis. *P<0.05, **P<0.01.

Next, we confirmed the association between NELL-1 and Wnt/β-catenin signalling in both OB and OC cells (Fig. 4). First, the M2-10B4 mouse bone marrow stromal cell (BMSC) line was treated with rhNELL-1 or WNT3A as a positive control. By three independent methods, rhNELL-1 induced a significant increase in Wnt/β-catenin (Fig. 4a–c). First, immunocytochemistry for active β-catenin showed increased staining (Fig. 4a). Western blot analysis for cytoplasmic and nuclear fractions demonstrated increased nuclear β-catenin (Fig. 4b,c). Finally, M2-10B4 cells expressing the TOPFLASH reporter system exhibited an increase in TCF/LEF1 activity (Fig. 4d). Next, we determined whether interference with Wnt/β-catenin signalling would impede rhNELL-1-induced OB differentiation. To answer this, two antagonists of Wnt signalling were used: DKK-1 (Fig. 4e) or XAV939 (Fig. 4f). Results showed that both Wnt antagonists inhibited rhNELL-1's induction of Runx2. We next determined whether rhNELL-1 induced Wnt/β-catenin signalling in OC precursor cells, using the mouse OC line RAW264.7 (Fig. 4g–j). As assessed using immunocytochemistry, nuclear β-catenin accumulation, and gene markers of Wnt/β-catenin signalling, rhNELL-1 showed a similar induction of Wnt signalling activity in RAW264.7 cells. In summary, rhNELL-1 protein activates Wnt/β-catenin signalling in both OB and OC cell types *in vitro*. Moreover, NELL-1's induction of OB differentiation is dependent on intact Wnt/β-catenin signalling.

We have previously determined that NELL-1 binds to the surface receptor integrin β1, and that this interaction is necessary for NELL-1's positive regulation of OB proliferation and adhesion^22^. Hasebe *et al*.^31^ subsequently verified that NELL-1 binds to integrin β1 and integrin α3. Here we confirmed these findings by thermal shift assays using integrin α3β1 with or without NELL-1 (Supplementary Fig. 4). In order to predict potential binding sites between NELL-1 and integrin β1, we next employed computational modelling to determine likely sites of protein interaction (Supplementary Fig. 4b–d). Integrins are α/β heterodimeric cell surface receptors that control numerous cellular processes, including OB and OC proliferation, differentiation and activity^32^. Furthermore, integrin receptor complexes containing integrin β1 activate Wnt/β-catenin signalling via integrin-linked kinase signalling and modulation of Glycogen synthase kinase 3 beta (GSK3β) phosphorylation^33^. Therefore, we assayed whether integrin β1 was required for NELL-1 activation of Wnt/β-catenin signalling (Fig. 4k–p). Small interfering RNA (siRNA)-mediated knockdown of integrin β1 inhibited rhNELL-1's activation of Wnt/β-catenin signalling, both in OB and OC precursor cell lines. Thus, integrin β1 is required for NELL-1 activation of Wnt/β-catenin signalling in both OB and OC cell types.

Subsequently, we sought to extend these findings to the human patient. For this purpose, human (h)BMSCs were harvested from patients with or without a clinical history of osteoporosis (Supplementary Table 2, Supplementary Fig. 5). NELL-1 signalling was manipulated by adenoviral *Nell-1* (Ad-Nell-1) or control (Ad-GFP). Ad-Nell-1 increased OB differentiation in hBMSC derived from either osteoporotic or nonosteoporotic samples. Further, Ad-Nell-1 treatment resulted in increased Wnt/β-catenin signalling activity in nonosteoporotic and osteoporotic hBMSC, as shown by gene markers and nuclear accumulation of β-catenin.

In summary, NELL-1 activates Wnt/β-catenin signalling in OB and OC precursor cells, in a process requiring integrin β1. Moreover, NELL-1 signalling activates Wnt/β-catenin signalling in human cells, from either nonosteoporotic or osteoporotic patients.

### NELL-1 increases bone formation in osteoporotic sheep

To translate NELL-1's osteogenic function into a clinically relevant large animal model, local surgical delivery of rhNELL-1 was performed in the sheep spine, which have similar dimensions, mineral content and collagen composition to that of humans^34^,^35^,^36^,^37^. Induction of osteoporosis was achieved using ovariectomy (OVX), glucocorticoid administration and a low-calcium and low-vitamin D diet (Supplementary Fig. 6a). Similar to human osteoporosis, lumber spines are significantly compromised and are prone to compression fracture. Local surgical delivery of rhNELL-1 was performed to L1, 3 and 5. rhNELL-1 protein was injected into the cancellous bone of the vertebral body, after lyophilization on β-tricalcium phosphate and using a hyaluronic acid carrier (Supplementary Table 3 for injection composition).

**Figure 5:**
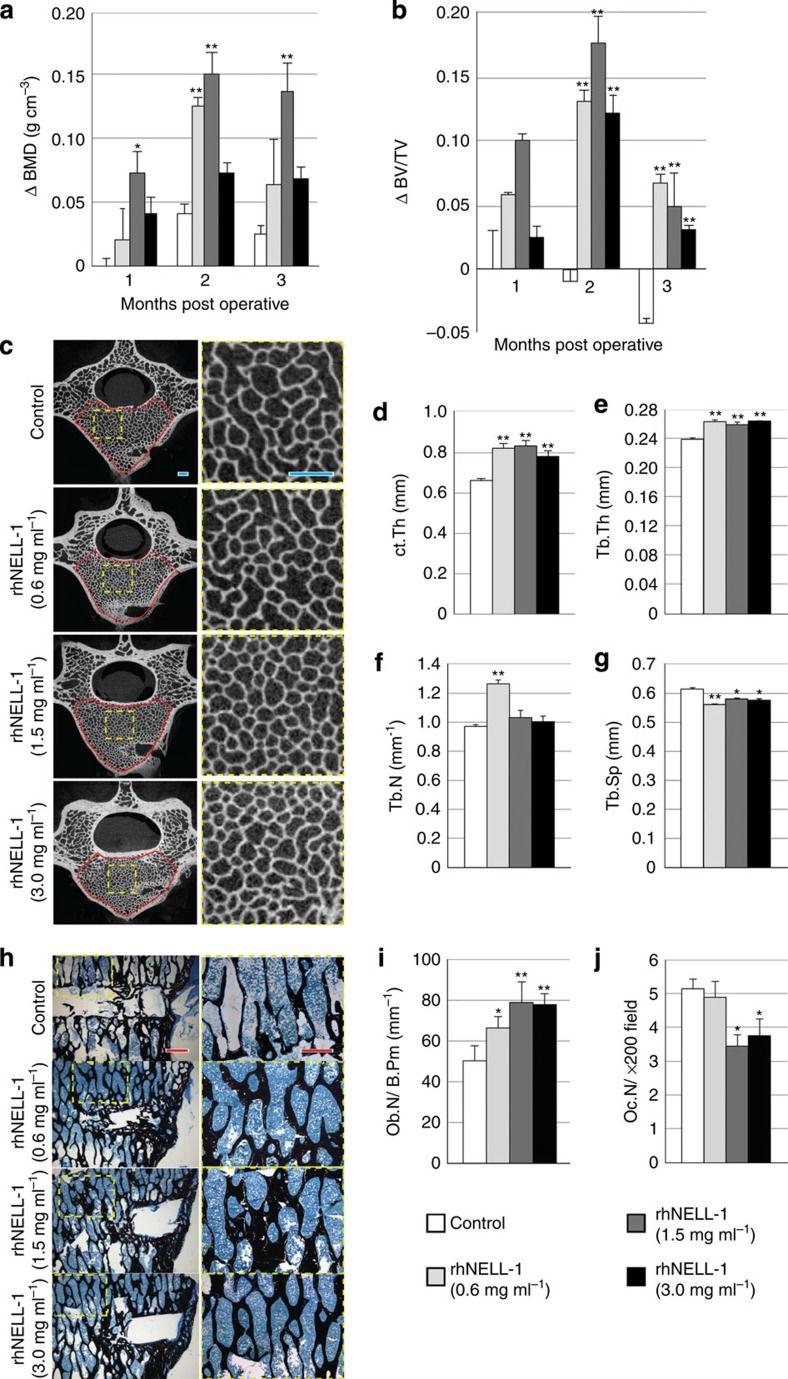
RhNELL-1 intravertebral injection increases bone formation in osteoporotic sheep. Recombinant human (rh)NELL-1 protein or vehicle control was injected into the lumbar vertebral bodies of osteoporotic sheep. RhNELL-1 was lyophilized on β-tricalcium phosphate (β-TCP) particles, which has been previously shown to increase the stability of rhNELL-1 *in vivo*^25^. Composition of the injected materials can be found in Supplementary Table 3. (**a**) Absolute change in trabecular BMD among control- and rhNELL-1-treated vertebrae by CT quantification of individual vertebral bodies (*N*=9 control-treated vertebrae, *N*=3 vertebrae per treatment dosage). (**b**) Percent change in the bone volume/tissue volume (BV/TV) among control- and rhNELL-1-treated vertebrae by CT quantification of individual vertebral bodies. All quantified values are normalized to month 1, control treatment data (*N*=9 control-treated vertebrae, *N*=3 vertebrae per treatment dose). (**c**) Axial sections of high-resolution CT images. Yellow dashed boxes indicate the area of high magnification on right. Red outlines indicate ROI for trabecular analyses. (**d**) Ct.Th as determined with microCT quantification, using the cortex ipisilateral to rhNELL-1 injection (*N*=162 control-treated measurements, *N*=54 measurements per treatment dose). (**e–g**) Trabecular analyses, including Tb.Th, Tb.N and Tb.Sp (*N*=9 control-treated vertebrae, *N*=3 vertebrae per treatment dose). (**h**) Representative images of Von Kossa–MacNeal's Tetrachrome (VKMT) staining trabecular bone adjacent to the injection tract. Yellow dashed boxes indicate the area of high magnification on right. (**i**) Ob.N per B.Pm (N=10, 9, 9 and 9 images, respectively). (**j**) Oc.N per × 200 field (N=9, 10, 9 and 9 images, respectively). Red scale bars, 0.5 mm; blue scale bars, 1.0 mm. Data points indicate the means, while error bars represent one s.e.m. *In vivo* experiments were performed without replicate, unless otherwise described. Parametric data were analysed using an appropriate Student's *t*-test or a one-way ANOVA, followed by a *post hoc* Tukey's test. Nonparametric data were analysed with a Mann–Whitney *U*-test or a Kruskal–Wallis one-way analysis. *P<0.05, **P<0.01 in comparison with vehicle control. See Supplementary Fig. 6 for additional details of sheep experimentation and analyses.

Live CT scans performed monthly after rhNELL-1 injection showed a significant increase in BMD and bone volume in rhNELL-1-treated vertebrae (Fig. 5a,b). Moreover, high-resolution microCT imaging and quantification showed increased Cortical bone Thickness (Ct.Th) and increased trabecular bone density in rhNELL-1-treated vertebrae (Fig. 5c–g). Histological examination confirmed a significant anabolic response to rhNELL-1 injection (Fig. 5h), quantified by histomorphometric analysis of cortical and trabecular bone measurements in the peri-injection area (Supplementary Table 4). Bone distant from the injection site was analysed, showing a similar anabolic response (Supplementary Fig. 6d–g). We next examined the effects of rhNELL-1 on OB and OC number. Consistent with our *in vitro* observations, rhNELL-1 significantly increased Ob.N and either reduced or had no effect on Oc.N (Fig. 5i,j). In summary, local rhNELL-1 delivery had significant and sustained bone-forming effects in osteoporotic sheep, observed in both cortical and cancellous bone, and accompanied by an increased OB:OC ratio.

### Systemic NELL-1 increases bone formation

**Figure 6:**
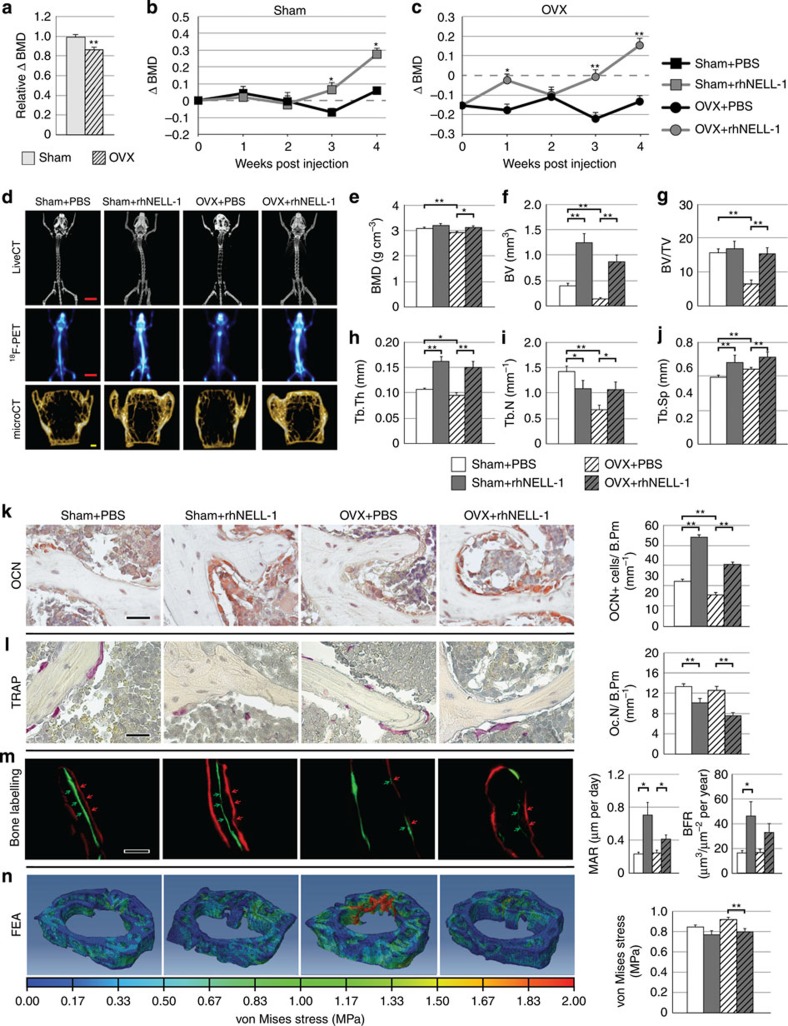
RhNELL-1 intravenous injection increases bone formation in osteoporotic mice. (**a**) Induction of osteoporosis was performed by OVX and ensuing bone loss over a 5-week period, confirmed by DXA analysis of the lumbar vertebrae (*N*=13 and 15 mice, respectively). Confirmation of OVX was also performed post mortem by demonstration of uterine atrophy (Supplementary Fig. 7b,c). (**b,c**) RhNELL-1 was next administered by tail vein injection (1.25 mg kg^−1^ q48 hr) over a period of 4 weeks, and compared with PBS control treatment (N=6, 7, 6 and 9 mice, respectively). DXA analysis of the lumbar vertebrae was performed weekly. (**d**) Representative images after 4 weeks of rhNELL-1 treatment, including live CT, ^18^F-PET and microCT. MicroCT indices have been compared with the published norms to ensure accuracy of analysis and reporting^59^,^60^,^61^. (**e–i**) MicroCT quantification after 4-week treatment, including (**e**) BMD, (**f**) BV, (**g**) BV/TV, (**h**) Tb.Th, (**i**) Tb.N and (**j**) Tb.Sp (*N*=6, 7, 6 and 9 mice, respectively). (**k**) Osteocalcin (OCN) immunohistochemical staining, and quantification of OCN+ bone-lining cells per B.Pm (*N*=18, 23, 25 and 30 images, respectively). (**l**) TRAP staining and quantification of TRAP+, multinucleated, bone-lining cells per B.Pm (*N*=28, 34, 28 and 40 images, respectively). (**m**) Calcein/Alizarin red complex on bone labelling and quantification of mineral apposition rate (MAR) and BFR. Red and green arrows highlight the space between fluorochrome labels (*N*=3 mice and 6 measurement fields per treatment group). (**n**) FEA and quantification of von Mises stress. Red colour indicates areas of high stress (*N*=6 per treatment group). Black scale bars, 25 μm; red scale bar, 10 mm; yellow scale bar, 100 μm. Data points indicate the means, while error bars represent one s.e.m. *In vivo* experiments were performed without replicate, unless otherwise described. Parametric data were analysed using an appropriate Student's *t*-test or a one-way ANOVA, followed by a *post hoc* Tukey's test. Nonparametric data were analysed with a Mann–Whitney *U*-test or a Kruskal–Wallis one-way analysis. *P<0.05, **P<0.01 in comparison with PBS control, unless otherwise indicated.

While the local effects of rhNELL-1 on bone formation have been established in other models^24^,^25^,^26^,^27^, the effects of systemic rhNELL-1 administration are entirely unknown and represent a broader impact for the treatment of osteoporosis. Systemic delivery was achieved by intravenous injection of rhNELL-1 in either nonosteoporotic or OVX-induced osteoporotic mice (Fig. 6, Supplementary Fig. 7). As expected, OVX induced a loss in the mean BMD, observed over a 5-week period (Fig. 6a). Next, we examined the pharmacokinetics of systemic rhNELL-1 (Supplementary Table 5). The elimination half-life of systemic rhNELL-1 was found to be 5.52 h (Supplementary Fig. 7a); thus, for sustained treatment rhNELL-1 was injected every 48 h. In comparison with control and over a 4-week period, results showed that systemic rhNELL-1 treatment induced significant bone formation in both non-OVX and OVX mice (Fig. 6b–n). Analysis of the lumbar vertebrae showed rhNELL-1-induced bone formation by weekly DXA (Fig. 6b,c), live CT/^18^F-PET imaging (Fig. 6d) and high-resolution microCT reconstruction and quantification (Fig. 6d–j). Histologic analyses were performed, including assessment of OB and OC markers (Fig. 6k,l and Supplementary Fig. 7d–g). In agreement with our prior *in vitro* and *in vivo* observations, systemic rhNELL-1 administration showed both pro-OB and anti-OC effects, with an increase in Ob.N and reduction in Oc.N. This was accompanied by reduced RANKL and increased osteoprotegerin (OPG) protein expression with rhNELL-1 administration, as observed using immunohistochemistry (Supplementary Fig. 7f,g). Bone labelling and dynamic histomorphometric analyses demonstrated a significant increase in mineral apposition rate and bone formation rate (BFR) with rhNELL-1 administration (Fig. 6m). Moreover, computer-simulated biomechanical (FEA) testing demonstrated increased bone strength with rhNELL-1 treatment, in both sham and OVX conditions (Fig. 6n). Importantly, no adverse effects were observed with rhNELL-1 in animal morbidity or mortality across the study period. As an external validity, formal 5-day repeat intravenous toxicity testing in mice showed no adverse effects (Supplementary Tables 9 and10; performed by Pacific Bioloab, Hercules, CA). Thus, NELL-1, an osteoinductive protein that activates Wnt/β-catenin signalling via integrin β1, can be a safe, systemic therapy to improve osteoporotic bone quality, tipping the balance in favour of bone anabolism over bone resorption.

## Discussion

In summary, we report that *Nell-1* deficiency in aged mice results in an osteoporotic phenotype, characterized by increased bone fragility, reduced Wnt/β-catenin signalling and reduced OB:OC ratio in terms of cell number and activity. On the cellular level, NELL-1 exerts its effects through interaction with integrin β1, and subsequent positive regulation of Wnt/β-catenin signalling. Finally, rhNELL-1 increases endocortical and cancellous bone in osteoporotic animal models, via either local or systemic administration. We conclude that NELL-1 activates Wnt/β-catenin signalling via integrin β1, uncouples OB:OC activity, may play a protective role against bone loss and represents a new anabolic and anti-OC pharmacotherapeutic agent.

First, we have found that NELL-1 differs from most other osteoinductive molecules in its negative effects on OC bone resorption. Typically, anabolic agents also produce a secondary osteoclastogenic response. In contrast, we observed NELL-1 to have inhibitory effects on OCs, with decreased OC number and activity—much like anti-Wnt inhibitor therapies. Our studies suggest that NELL-1's OC inhibition may be direct and/or indirect. NELL-1 directly activates Wnt/β-catenin signalling in both OB and OC cell types. Wnt/β-catenin signalling in OBs is well described to induce OB-derived expression of the major OC inhibitor OPG, and alteration of the balance between OPG and RANKL, thereby indirectly inhibiting OC activity^38^,^39^,^40^,^41^. In addition, Wnt/β-catenin signalling activation in OC precursors has antiosteoclastogenic effects, independent of OB-elaborated OPG^42^. Therefore, NELL-1's positive effects on bone formation and negative effects on bone resorption may be explained both by activation of Wnt/β-catenin signalling in OB and OC precursors, respectively. The extent to which the anti-OC effects of NELL-1 are similar or different to anti-Wnt inhibitor therapies, such as anti-SOST antibodies, is yet to be determined.

Second, we have shown that NELL-1 activation of Wnt/β-catenin in OB and OC cell types requires expression of integrin β1. Our research group recently identified integrin β1 as the only known cell surface receptor for NELL-1, and that NELL-1's effects on OB attachment required integrin β1 expression^22^. Another research group subsequently verified that the C-terminal region of NELL-1 binds to integrin α3β1 (ref. 31). Integrins have documented roles in OB cell attachment, proliferation and differentiation^43^,^44^ and, likewise, known functions in OC adhesion and regulation of cytoskeletal organization for OC-resorptive function^45^. In particular, integrin αvβ3 is highly expressed in OC and mediates bone extracellular matrix attachment^45^. Although the specific effects of integrin β1 agonists have not been examined, integrin β1 ablation led to reduced OC-resorptive capacity^46^. Our findings of NELL-1 activation of Wnt/β-catenin signalling in OCs via integrin β1 may represent a new and unexplored function of integrins in OCs^33^. In summary, our observations of NELL-1 interacting with integrin β1 to exert its cellular and tissue-level effects are overall in agreement with the known roles of integrin β1 in bone biology.

Finally, we have found that NELL-1 has potential dual uses as both a local bone-forming growth factor as well as a systemic osteogenic factor for osteoporosis. The present study advances our knowledge of the local bone-forming effects of NELL-1 to a sheep osteoporotic model. Therapeutic options for bone graft in the osteoporotic patient are limited. For example, autograft bone is less effective^47^ and donor-site fracture is more common^48^. With similar drawbacks, BMP2-based substitutes induce direct stimulation of bone resorption^49^,^50^, leading to vertebral subsidence or collapse^51^. With these limitations of current bone graft substitutes in the osteoporotic population, NELL-1 may be a future bone graft substitute well suited for the osteoporotic patient.

We have also demonstrated the potential therapeutic application of systemic NELL-1 delivery for the reversal of osteoporosis. NELL-1 possesses several practical and theoretical benefits over other systemic therapies for osteoporosis. First, as we observed, NELL-1 has dual anabolic and anti-OC properties. Second, NELL-1 has documented tumour-suppressive properties and its expression is lost in several carcinomas^52^,^53^. This lies in contrast to PTH, whose clinical use is limited by the risk of osteosarcoma as suggested by rat studies^54^. Finally, NELL-1 has an excellent safety profile. Mice with constitutive *Nell-1* overexpression have a normal lifespan, and are without abnormalities excepting the skeleton^20^. Similarly, formal intravenous NELL-1 toxicity testing found no pathologic or biochemical abnormalities. Although the kinetics of unmodified NELL-1 protein involve rapid elimination, structural modification of NELL-1 may allow for sustained therapeutic serum levels. However, NELL-1 has known functions in neurogenesis^55^, chondrogenesis^56^ and vasculogenesis^57^ —and a thorough study of these off-bone effects must be instituted before consideration of NELL-1 as an osteoporotic therapy.

In summary, we demonstrate that NELL-1 deficiency induces age-related osteoporosis in a rodent model. Mechanistically, our data suggest that NELL-1 modulates OB and OC activities through Integrinβ1-binding and subsequent Wnt/β-catenin signalling activation. Most importantly, the therapeutic impact of our large and small osteoporotic animal studies demonstrates how NELL-1 may be used as a new anabolic, anti-OC treatment for osteoporosis in both local and systemic intervention. Finally, demonstration of the ability of systemic NELL-1 to activate Wnt/β-catenin signalling will provide fundamental insights relevant to the development of NELL-1-based therapies for the treatment of multiple bone pathologies.

## Methods

### Animal care

All animals were cared for according to the institutional guidelines set by the Chancellor's Animal Research Committee of the Office for Protection of Research Subjects at the University of California, Los Angeles as well as the UCLA Office of Animal Research Oversight. Matings were carried out overnight and females were examined for the presence of vaginal plugs, E 0.5 dpc (days post coitum). Animal groups were of mixed gender unless otherwise stated. CD-1 wild-type mice were obtained from Charles River for bone marrow OB precursor, OC precursor and calvarial bone isolation studies. B6 mice were obtained from Charles River for OVX studies. Sprague–Dawley rats were used for Nell-1 expression studies. Heterozygote carriers of the *Nell-1*^+/6R^ gene were provided by the Mammalian Genetic Research Facility at the Oak Ridge National Laboratory and were transferred with permission of the Chancellor's Animal Research Committee. The *Nell-1*^6R/6R^ mouse genotypes were identified from DNA extracted from clipped tails of mutant and wild-type mice. The extracted DNA was amplified using microsatellite primers D7Mit 315-L; 5′-TGATAACAAAACAGTCAGTATGAAGC-3′, D7Mit 315; 5′-RCTGATCCATCTGTATGATGTTACTTG-3′. All mice were housed in the light- and temperature-controlled UCLA vivarium, and provided water and feed *ad libitum*. Sheep were cared for at the Colorado State University according to the Veterinary Teaching Hospital institutional guidelines. All sheep were fed a grass/alfalfa mix hay and provided water and feed *ad libitum*. Whenever possible, animals were randomized with even distribution across treatment groups (including data presented in Figs 5 and 6).

### Murine radiographic analyses

Murine samples were analysed by DXA, live microCT/^18^F-PET and high-resolution microCT. DXA was performed as previously described on live *Nell-1*^+/+^ and *Nell-1*^+/6R^ littermates as follows. Scans were performed under isoflurane sedation using a Lunar PIXImus II Densiometer (GE Healthcare, Piscataway, NJ). BMD and bone mineral content (BMC) were calculated using a rectangular region of interest (ROI) encompassing the lumbar spine, or the distal left femur. Analyses were performed on 18-month-old mice; *N*=12 *Nell-1*^+/+^ and 16 *Nell-1*^+/6R^ mice were used for DXA analyses. Unless otherwise stated, for all studies utilizing *Nell-1*^+/6R^ mice, the entire colony of the studied age and gender was included for analysis. No exclusion criteria were established. Live computed tomography/positron emission tomography (CT/PET) studies were performed as follows. Fluoride ion was produced using ^18^O-water and proton bombardment using an RDS cyclotron (Siemens Preclinical Solutions). ^18^F-Fluoride ion was produced at specific activities of ∼37 TBq mmol^−1^. Using an animal research committee (ARC)-approved isolated imaging chamber, all animals underwent CT/PET scanning. Mice were injected with ^18^F-Fluroide ion via tail vein using a 27-gauge needle. Animals were positioned in a multimodality, portable isolated bed system. Whole-body scans were performed with a 10-min acquisition time using a microPET FOCUS 200 system (Siemens Preclinical Solutions). Immediately afterwards, a noncontrast-enhanced microCT study using a microCAT II (Siemens Preclinical Solutions) imaging system was used to scan animals with a 20-min acquisition time. PET scan images were reconstructed using filtered backprojection and an iterative three-dimensional (3D) reconstruction algorithm (maximum *a posteriori*). MicroCT images were created using Fledkamp reconstruction at 200-μm resolution. ^18^F-Flouride and CT data were analysed and quantified with AMIDE (A Medical Image Data Examiner). To quantify the BMD and ^18^F uptake, a cylindrical ROI was drawn encompassing a single lumbar vertebrae of set dimensions. Data for CT/PET images were compiled and analysed by three independent, blinded reviewers. *N*=8 mice per genotype were analysed at 1 and 6 months of age, *N*=11 *Nell-1*^+/+^ mice and 12 *Nell-1*^+/6R^ mice were analysed at 18 months of age. For PET, *N*=6 mice per genotype were analysed at 18 months of age. High-resolution, post-mortem microCT scanning and analysis were performed on neonatal whole mice (*N*=10 mice per genotype) and aged mouse spines (*N*=12 *Nell-1*^+/+^ and 19 *Nell-1*^+/6R^ individual vertebrae per genotype). Samples were harvested, formalin-fixed and imaged using high-resolution microCT (Skyscan 1172F, Skyscan, Belgium) at an image resolution of 17.8–28.2 μm and analysed using DataViewer, Recon, CTAn and CTVol softwares provided by the manufacturer. For neonatal spine CT data analysis, the ROI included the entire volume lumbar spine to quantify BMD and bone volume/tissue volume. Trabecular analysis was performed on aged mouse spine specimens, for which ROIs were drawn to include each individual lumbar vertebral body excluding the cortical bone. Reconstructions were performed using the Osirix software, using coronal cross-sectional images with a 0.25-μm width. All quantitative and structural morphometric data use nomenclature described by the American Society for Bone and Mineral Research Nomenclature Committee^58^. MicroCT indices were compared with the published norms to ensure accuracy of analysis and reporting^59^,^60^,^61^. Whenever possible, all radiographic studies were performed and quantified in a blinded manner. *Nell-1*^+/+^ and *Nell-1*^+/6R^ littermates look nearly identical, with the exception of a variably lighter coat colour among *Nell-1*^+/6R^ mice. However, unblinding of radiographic technicians would have minimal potential for the introduction of bias, as all radiographic studies were highly routinized. ROI construction was performed in a completely blinded manner. However, a visually osteopenic skeleton among *Nell-1*^+/6R^ mice in theory presented a potential for unblinding.

### Nuclear magnetic resonance

NMR was used to determine total body fat mass and lean mass on live *Nell-1*^+/+^ and *Nell-1*^+/6R^ littermates as follows. A Bruker Minispec was used with software from Echo Medical Systems (Houston, TX). In total, 12 *Nell-1*^+/+^ and 16 *Nell-1*^+/6R^ mice were used for NMR analyses. All mice were weighed before NMR analysis.

### Histologic analyses

For histology, all tissues were fixed in 10% PBS-buffered formalin. Mouse samples were decalcified in 19% EDTA and embedded in paraffin. Five-micron-thick sections were made and stained with haematoxylin and eosin, Masson's Trichrome, Aniline Blue and TRAP staining (Sigma-Aldrich) as per the standard protocols. Analysis of Masson's Trichrome and Aniline Blue staining was performed to demonstrate the degree of mineralization between samples. Histological specimens were analysed using the Olympus BX51 microscopes and images acquired using MicroFire digital camera with the Picture Frame software (Optronics, Goleta, CA). Indirect immunohistochemistry was performed using 3-amino-9-ethylcarbazole (AEC) as chromogen, with primary antibodies listed in Supplementary Table 6. Histomorphometric analysis was performed using Adobe Photoshop. Measurements included bone area (B.Ar), percentage bone area (% B.Ar), B.Pm, cortical width and trabecular analyses (trabecular bone width, trabecular bone number, trabecular bone spacing). Numbers of quantified images for each analysis are as follows: B.Ar (*N*=29 *Nell-1*^+/+^ and 26 *Nell-1*^+/6R^ images), % B.Ar (*N*=29 *Nell-1*^+/+^ and 26 *Nell-1*^+/6R^ images) and B.Pm (*N*=30 *Nell-1*^+/+^ and 29 *Nell-1*^+/6R^ images). Ob.N was assessed by per high-power field of Masson's Trichrome staining (*N*=20 *Nell-1*^+/+^ and 20 *Nell-1*^+/6R^ images analysed; *N*=18–30 images per treatment group analysed for Fig. 6), while Oc.N was assessed per high-power field of TRAP staining (*N*=26 *Nell-1*^+/+^ and 39 *Nell-1*^+/6R^ images analysed; *N*=28–40 images per treatment group analysed for Fig. 6). Cytomorphologic definition of an OB required bone-lining cells with a single round to ovoid nuclei and fairly abundant cytoplasm. Characteristic cell morphology with three or more nuclei was used to define Oc.N. Analyses of immunohistochemical staining are either reported as the number of immunoreactive cells per B.Pm, or semiquantitative measurement of relative total immunoreactivity. Semiquantitative measurements were performed using the magic wand tool in Adobe Photoshop.

### Serum studies

ELISA-based serum studies were performed using serum samples as per the manufacturer's instructions. Briefly, *Nell-1*^+/+^ and *Nell-1*^+/6R^ littermates were bled via the retro-orbital sinus at 0900 hours on the same day after overnight fasting. Serum was collected via centrifugation and stored before examination of serum PINP, serum TRAP5b and serum CTX. All assays were obtained from ImmunoDiagnostic Systems Inc. and performed according to the manufacturer's instructions. For serum PINP, a rat/mouse enzymeimmunoassay was used (*N*=19 and 17 mice for *Nell-1*^+/+^ and *Nell-1*^+/6R^ mice, respectively). For serum CTX, a RatLaps Enzymeimmunoassay was used (*N*=12 and 22 mice for *Nell-1*^+/+^ and *Nell-1*^+/6R^ mice, respectively). For serum TRAP5b, a MouseTRAP solid-phase immunofixed enzyme activity assay was used (*N*=10 and 9 mice for *Nell-1*^+/+^ and *Nell-1*^+/6R^ mice, respectively; kits listed in Supplementary Table 7). Gene expression was performed after microdissection of lower lumbar vertebral bodies, following by mRNA extraction and quantitative real-time (RT)-PCR. *N*=3 samples (Fig. 1c) or 4 samples (Fig. 3c) per genotype were used for analysis. Primers are listed in Supplementary Table 8.

### Biomechanical analyses

For biomechanical testing, two methods were independently used: the BioDent Reference Point Indenter and computerized simulation (finite element analysis, FEA). Analysis of the S1 vertebral body was performed on all mice. Lumbar vertebrae could not be used, as the spines in the *Nell-1*^+/6R^ mice were too fragile for analysis. The indenter was placed on the dorsal periosteal surface for measurement acquisition. The following settings were used: 2*N* indentation force, 2 indentations per second and 10 indentations per measurement. Total indentation distance was calculated by measuring the maximum indentation distance achieved during a measurement. Indentation distance increase (IDI) was calculated by measuring the difference between the depths reached at peak force during the first indentation cycle and last indentation cycle. Unloading stiffness is calculated by evaluating the top portion of the unloading section of the force displacement curve. *N*=3 mice per genotype, with *N*=6 measurements per mouse used for indentation studies.

FEA (biomechanical) was performed using microcT images converted to DICOM files using the SKyScan Dicom Converter software (DicomCT application, Skyscan 1172F, Skyscan). Tetrahedral 3D mesh models were created using an VOI of either the lumbar spine (level 4) using the ScanIP software (Simpleware Limited). A constant thickness of 0.54 mm was used for both VOIs. FEAs were performed using the ABAQUS software (Dassault Systèmes) with boundary conditions set as encastre, constrained in all directions. Next, we applied a uniform compressive pressure of 0.5 MPa on the superior surface of the Volume of interest (VOI). The von Mises stress experienced and total strain energy of the samples were analysed. *N*=8 mice per genotype for FEA analyses.

### Dynamic histomorphometric analyses

For bone fluorescent-labelling studies, mice were injected intraperitoneally with calcein (20 mg kg^−1^) and alizarin red complexon (50 mg kg^−1^) at 9 and 2 days before being killed, respectively. Lumbar vertebrae were dissected, fixed in 70% ethanol, dehydrated and embedded undecalcified in methyl methacrylate. Coronal sections at 5-μm thickness were analysed using the OsteoMeasure morphometry system (Osteometrics, Atlanta, GA, USA). For dynamic histomorphometry, the mineral apposition rate (μm per day), the distance between the midpoints of the two labels divided by the time between the midpoints of the interval were measured in unstained sections under ultraviolet light and used to calculate the bone formation rate with a bone surface referent (BFR/BS, μm^3 ^μm^−2^ per year). The bone formation rate per bone surface (BFR/BS) is the volume of mineralized bone formed per unit time and per unit bone surface. The fixed area and location at × 200 magnification of lumbar vertebral 4–5 (L4–5) was selected as the ROI (*N*=3 mice and 6 measurement fields per treatment group). All image acquisition and analyses were performed in a blinded manner.

### OB precursor culture and experiments

Mouse OB precursors were harvested by flushing the femoral marrow cavities and harvesting adherent cells on standard culture-treated plates. For isolation of *Nell-1*^+/+^ and *Nell-1*^+/6R^ OB precursors, cells were isolated from 18-month-old littermates (*N*=3 mice per genotype). For additional experiments using wild-type OB precursors, 3-month-old CD-1 animals were used. Proliferation and osteogenic differentiation and assessments were performed with or without recombinant human (rh)NELL-1 protein (100–300 ng ml^−1^). The osteogenic differentiation medium was constituted with 10 mM β-glycerophosphate and 50 μM ascorbic acid (Fisher Scientific, Pittsburgh, PA) in high-glucose DMEM, 10% fetal bovine serum (FBS), 1% penicillin/streptomycin (GIBCO, Invitrogen, Carlsbad, CA). To assess early to intermediate OB differentiation, ALP staining and quantification was performed in each case normalized to total protein content in sister wells. To assess bone nodule formation, Alizarin red staining and quantification was performed using CPC leaching and photometric quantification, normalized to total protein content. Quantitative real-time PCR was performed in triplicate wells per RNA isolate. Primers are listed in Supplementary Table 8. All experiments were performed in triplicate wells. Passage 2 OB precursors only were used for all assays. For all *in vitro* studies, technical execution of the experiments was performed in an unblinded manner, unless otherwise noted.

### OC precursor resorption experiments

Calvarial discs were obtained using a 2-mm punch biopsy from the mid-parietal bone of postnatal day 7, CD-1, wild-type mice. Discs were processed by gentle removal of periosteal and dural tissues and stored in 100% ethanol to decellularize the tissues at 4 °C. Mouse OC precursors were obtained by femoral and tibial marrow flushing. For isolation of *Nell-1*^+/+^ and *Nell-1*^+/6R^, OC precursors were isolated from 18-month-old littermates (*N*=3 mice per genotype used). For additional experiments using wild-type OC precursors, 3-month-old CD-1 animals were used. The product of total bone marrow flush was cultured in 25 ng ml^−1^ recombinant mouse M-CSF (R&D Systems, Minneapolis, MN) overnight. The nonadherent cells were then cultured on calvarial discs for resorption assays in phenol red-free αMEM media, 10% FBS, 25 ng ml^−1^ recombinant mouse Macrophage Colony Stimulating Factor (rmM-CSF) and 100 ng ml^−1^ recombinant soluble RANKL (R&D Systems), with or without rhNELL-1 (0–1200, ng ml^−1^). After 5 days, calvarial discs were harvested, washed in 1 × PBS and stained with 1% Toluidine Blue dye in dilute Sodium Borate (Fisher Scientific). Calvarial discs were photographed and quantification of intensity blue staining (indicating the presence of bone) was performed using the magic wand tool in Adobe Photoshop CS5 (*N*=9 and 11 samples for *Nell-1*^+/+^ and *Nell-1*^+/6R^ samples, respectively). Next, scanning electron microscopy was performed as using a Nova 230 microscope (FEI, Hillsboro, Oregon) in a low vacuum mode operated at 40 Pascal. Accelerating voltage was used at 10 kV with a working difference varying between 4.8 and 5.7 mm. Samples were analysed at a magnification of × 200. The MeX 3D software version 5.1 (Alicona Imaging Corporation, Austria) was used to analyse the mean resorption pit depth and the mean roughness of the calvarial discs. In order to measure the depth of the pits, several lines were drawn through each suspected pit at 30° intervals; the line that provided the largest difference between peak and valley was used, determining the distance in mm between the peak and valley measurements. Resorption pits were defined as having a minimum depth of 1.5 μm (*N*=9 measurements per genotype). Average roughness was measured by drawing six lines across each sample at 100-μm intervals using the MeX software (*N*=36 and 29 measurements for *Nell-1*^+/+^ and *Nell-1*^+/6R^ samples, respectively). The calculated roughness of each line was then used to calculate the mean roughness per treatment group. s.e.m. measurements were performed in a blinded manner.

### M2-10B4 and RAW264.7 cell culture

The M2-10B4 cell line, a clone derived from BMSCs from a (C57BL/6J X C3H/HeJ) F1 mouse, was purchased from American Type Culture Collection (ATCC no. CRL-1972, Lot no. 58696031, Manassas, VA) and used for experiments within 6 months of mycoplasma contamination testing. RAW264.7 cells were a kind gift from the laboratory of Dr Tintut. Cells were maintained in growth medium (RPMI 1640 supplemented with 10% FBS, 1 mM sodium pyruvate and 100 U ml^−1^ penicillin/streptomycin). RAW264.7 cells were cultured in αMEM+10% FBS. Wnt/β-catenin signalling was assayed using four methods: immunocytochemistry for active β-catenin, western blotting for nuclear β-catenin, TCF/LEF1 reporter activity (TOPFLASH) and qRT–PCR. Experiments used recombinant WNT3A as a positive control. For immunocytochemistry, M2-10B4 cells were seeded on Millicell EX Slides (PEZGS0816, Millipore) at 5 × 10^4^ cells per well in RPMI 1640+10% FBS for 24 h and serum-starved in RPMI 1640+1% FBS overnight. After 2-h treatment, cells were fixed using ice-cold acetone for 10 min. Anti-active β-catenin antibody (Millipore) was applied at a dilution of 1:200. The ABC complex (Vector Laboratories, Burlingame, CA) was applied to the sections following incubation with biotinylated secondary antibody (Dako). AEC substrate (Dako) was used as a chromogen, and the sections were lightly counterstained with haematoxylin. Photomicrographs were acquired using Olympus BX51 (× 200 magnification lens, UPLanFL, Olympus; *N*=4 wells per treatment group). For western blot analysis, nuclear and cytoplasmic protein was isolated using the NE-PER Nuclear and Cytoplasmic Extraction Kit (Thermo Scientific, Rockford, IL). Western blot analysis was performed using an antibody against β-catenin at a dilution of 1:1,000 (*N*=3 wells per treatment group). For TCF/LEF1 reporter activity, cells were transfected with 20 μg Super(16 × ) TOPFLASH (TCF/LEF1 Reporter Plasmid) and 1 μg *Renilla* Luciferase plasmids for 24 h and then seeded at 4.0 × 10^3^ cells per well in 96-well plates. Cells were starved in RPMI 1640+1% FBS overnight and then treated with rhNELL-1. Luciferase activity was measured 48 h after treatment using the Dual Luciferase Reporter Assay System (Promega, Madison, WI) as per the manufacturer's instructions (*N*=4 wells per treatment group).

### Lentiviral vector for runx2 reporter assay

The transduction plasmid for preparing lentiviral vector carrying the *Runx2* P1-EGFP expression cassette (*Runx2*-EGFP reporter) was prepared by substituting the CMV promoter in the pRRL-cPPT-CMV-X-PRE-SIN plasmid with the mouse *Runx2* P1 promoter. The mouse *Runx2* P1 promoter was obtained with PCR of mouse genomic DNA (forward primer: 5′-gcgaattactcgagagcagcactgttgctcagaa-3′; reverse primer: 5′-gcgaatgcccgggtcacacaatccaaaaaagc-3′). 293T cells were co-transfected with the transduction plasmids, the package plasmid pCMV-dR8.2-vprX and the envelope plasmid pCMV-VSVG. The viral vectors were collected at 2–4 days post transfection, filtered and concentrated, and the concentrations of viral vector were quantified by counting the core protein p24 using ELISA assay. For cell transfection, M2-10B4 cells were seeded in 24-well cell culture plates at 2 × 10^4^ cells per well 16 h before infection. Viral vectors with p24 counts of 0.4 μg were added to each well in 24-well plates. Three hours post infection, the viral vectors were washed away and fresh medium was added to the cultures. At 24 h post infection, rhNELL-1 was added into the culture media. Cells were trypsinized and collected 3 days post infection. Flow cytometry was performed to quantify green fluorescent protein (GFP) expression in the collected cells using a Cytomics FC500 cell sorter (Beckman Coulter, Brea, CA). Cells infected with mock vector were used as the negative control to establish gates. The percentages of GFP-positive cells were counted to quantify the expression of GFP in the infected cultures (*N*=4 wells per treatment). For select experiments, DKK-1 (100 ng ml^−1^) or XAV939 (1 μM) were used (*N*=4 wells per treatment group).

### siRNA experiments

RNA knockdown experiments were performed in M2-10B4 and RAW264.7 cells using chemically synthesized and annealed siRNA specific to integrin β1 (Santa Cruz, sc-35675), with *N*=3 wells per treatment group. Integrin β1 siRNA is a pool of four different siRNA duplexes: (A) Sense: 5′-GAACGGAUUUGAUGAAUGAtt-3′, Antisense: 5′-UCAUUCAUCAAAUCCGUUCtt-3′, (B) Sense: 5′-CAUUGGCUUUGGCUCAUUUtt-3′, Antisense: 5′-AAAUGAGCCAAAGCCAAUGtt-3′, (C) Sense: 5′-CAACCUGUUUACAAGGAAUtt-3′, Antisense: 5′-AUUCCUUGUAAACAGGUUGtt-3′, (D) Sense: 5′-UUUGGACACUGGUCCAUGUtt-3′, Antisense: 5′-ACAUGGACCAGUGUCCAAAtt-3′. Control siRNA was used (SC-37007) with sequence r(5′-UUCUCCGAACGUGUCACGU)d(TT)-3′. When cells reached 30% confluence, cells were transfected with 50 nM integrin β1 siRNA or nontarget negative control siRNA (Santa Cruz) using Lipofectamine RNAiMax (Invitrogen). Efficiency of knockdown was validated using western blot analysis.

### Human BMSC culture

Human BMSCs were obtained from *N*=5 patients, with or without a history of osteoporosis (see Supplementary Table 2 for a description of patient samples). Samples were obtained during a surgical procedure, predominantly for fracture or joint replacement surgery (see Supplementary Table 2), and with UCLA IRB exemption (IRB no. 11-000724). Samples were de-identified to protect the privacy of the patient. As no direct contact and no risk was poised to the patient, neither informed consent nor authorization was obtained, as per the University guidelines. Both nonosteoporotic and osteoporotic patients were without major medical comorbidities. Bone marrow tissues with bone chips were obtained and stored on ice during transport. These tissues were next digested with Type II collagenase (2 mg ml^−1^) under agitation and followed by Ficoll centrifugation. The mononuclear cells were separated and seeded into T-75 flasks for cell expansion. hBMSCs were obtained as adherent cells on standard culture-treated plates. Cells were expanded in αMEM+20% FBS on 10-mm cell culture plates. All experiments were performed in triplicate for each patient sample. To manipulate Nell-1 signalling, hBMSCs were transduced with Ad-Nell-1 or Ad-GFP at multiplicity of infection 50 pfu per cell. First, cells were either seeded for osteogenic differentiation assays (2 × 10^4^ cells per well) in 24-well plates. Osteogenic differentiation medium consisted of 50 μg ml^−1^ ascorbic acid and 10 mM β-glycerophosphate. Staining for AR was performed at 11 days of differentiation. Next, Wnt/β-catenin signalling activity was assayed on the gene and protein level. mRNA analysis was performed 3 days after transduction. For western blot analysis, cell lysate was collected 3 days after transduction. Nuclear and cytoplasmic fractions were extracted using the NE-PER Nuclear and Cytoplasmic Extraction Kit (Thermo Scientific). Western blot analysis was performed with anti-β-catenin (610153, BD Biosciences), anti-β-actin (sc-1616, Santa Cruz) and anti-H3 (AB8898, Abcam). Please see Supplementary Table 6 for specific antibody concentrations used. Quantification was performed using Image Pro. Taqman primers were used for human *NELL-1* (Hs00196243_m1) and human *GAPDH* (Hs99999905_m1). All experiments using human BMSC were performed in triplicate wells.

### Assessments of NELL-1/integrin β1 interaction

Confirmation of NELL-1 binding to integrin β1 was performed in two methods, including thermal shift assays and computational modelling. The thermal shift assay was performed using the 7,300 Real Time PCR System (Applied Biosystems, CA). Before use, the environmentally sensitive fluorescent dye SYPRO Orange stock solution in dimethylsulphoxide (5,000 × , Sigma) was diluted 1:125 in PBS. The samples were prepared in a 96-well plate in triplicate containing 2 μg NELL-1 and/or 1 μg integrin α3β1 (R&D Systems, 2840-A3-050) and freshly diluted SYPRO orange in PBS buffer (0.01 M, pH 7.4) with the total volume of 15 μl, and then the plate was sealed with optical quality sealing tape and centrifuged at 4,000 r.p.m. for 2 min. The fluorescent intensity was monitored as the plate was heated from 25 to 89 °C in an increment of 1 °C min^−1^. Second, computational modelling was performed between NELL-1 and the extracellular domain of integrin β1 based on RosettaDock. The three top-scoring models of NELL-1 to integrin β1 predicted by docking simulation are depicted in Supplementary Fig. 4.

### TOPGAL mouse intrafemoral Ad-Nell-1 injection

Wnt reporter TOPGAL mice were injected by intrafemoral injection with Ad-Nell-1 (5 × 10^12 ^pfu ml^−1^) or Ad-CMV-Null control (Vector BioLabs, Philadelphia, PA). Mice were randomized to each treatment group. Block randomization was used to ensure equal sample sizes. A percutaneous, infrapatellar approach was used, with a 26-gauge needle, and a 10-μl total injection volume. Animals were killed 1 and 2 weeks post injection for the analysis of β-galactosidase expression within the marrow space. For histological analysis, whole-mount X-gal staining was performed 1 week post injection followed by paraffin embedding and examination of serial sections. *N*=3 mice per treatment group. For quantification, flow cytometry was performed 2 weeks post injection. Bone marrow cells were flushed from the femur. Next, the femoral shaft was finely minced and the bone chips were digested with collagenase II (3 mg ml^−1^) for 2 h. The resultant cells from both bone marrow flush and cell digestion were combined, pelleted and washed twice with PBS+1% FBS. β-galactosidase substrate FDG (Invitrogen F1179) was then added to cells under hypotonic shock for 1 min and run for flow cytometry (LSRII, BD Bioscience). *N*=4 mice per treatment group.

### Mouse OVX and rhNELL-1 intravenous injection

OVX was performed on B6 mice with age-matched Sham controls at 12 weeks of age, using a 5-mm dorsal incision. Anaesthesia was performed with Isoflurane (3–5% for induction and 1.5–2% for maintenance). Pre-operative analgesia (Buprenorphine) was given once before surgery and twice daily for 2 days postoperatively. Five weeks post OVX, rhNELL-1 delivery by intravenous injection was instituted, by lateral tail vein injection using a 25-gauge needle. Animals were assigned to treatment groups by simple randomization. Drug administrators were completely blinded as to the treatment groups. The dosage of rhNELL-1 (1.25 mg kg^−1^) was obtained from pilot studies examining a wide range of doses. PBS served as vehicle control. Each injection consisted of a total 0.1 ml solution, and the injection was given every 48 h for the study period. Analyses were as per above, including DXA, live CT/PET, high-resolution microCT and quantification, histology and immunohistochemical staining. Animal numbers for each analysis were as follows (Sham Control, Sham rhNELL-1, OVX Control, OVX rhNELL-1): DXA (6, 7, 6 and 9 mice), high-resolution microCT (5, 5, 6, 9 mice), Osteocalcin quantification (18, 23, 25 and 30 images), TRAP quantification (28, 34, 28 and 40 images) and FEA (*N*=6, 7, 6, 9 mice). ROIs for analysis included the lumbar vertebrae. MicroCT indices were compared with the published norms to ensure the accuracy of analysis and reporting^59^,^60^,^61^. In addition, post-mortem analysis of uterus weight was obtained to confirm OVX. In select experiments, fluorescein isothiocyanate conjugation was employed to determine the pharmacokinetics of rhNELL-1 systemic delivery.

### Sheep experiment preparation and surgery

Osteoporosis was induced through OVX, controlled diet and steroid induction in eight adult ewes. Post OVX, three intramuscular injections of 500 mg methylprednisolone acetate (Depomedrol) were administered at 3-week intervals starting 2 weeks post operation. Special low-calcium and low-vitamin D osteoporosis diets were formulated in cooperation with Purina LabDiet and were fed to the sheep for 8 months post OVX. On the basis of pre-established inclusion criteria, of eight sheep in total, the six sheep with highest response to osteoporotic induction were used for further study. Successful induction of osteoporosis was confirmed using DXA imaging and quantification. The contents of material for sheep intrabody vertebral injection are in Supplementary Table 3, and included hyaluronic acid, β-tricalcium phosphate, with or without rhNELL-1 protein. Block randomization was used to ensure equal treatment group sizes. Surgeons were completely blinded to the treatment group. As injection contents appeared visually identical between groups, no unblinding of the operating surgeons was observed. Surgery was performed 4 months post OVX on *N*=6 osteoporotic sheep. A ventrolateral incision was made for a retroperitoneal approach. Induction of anaesthesia was performed by Valium or Midazolam or propofol along with ketamine through a venous catheter placed in an ear vein. Following induction, animals were intubated and transferred to Isoflurane in oxygen inhalant titrated to maintain a plane of surgical anaesthesia. Dissection was carried out to expose the cranial aspect of the L1-L5 vertebral bodies. A 3.2-mm unicortical drill hole was created on the left lateral aspect of the vertebral body. An introducer with stylet that perfectly fitted the drillhole was then inserted, ensuring an airtight, watertight seal. The stylet was then removed, and using a luer lock syringe the implant material injected into the introducer, and the stylet reinserted so as to push all materials into the cancellous bone of the vertebral body. No leakage of injection contents was observed. Bone wax was used to seal the drill hole. Three different vertebral levels were instrumented per sheep (L1, L3 and L5). Three sheep were injected with the control vehicle and three sheep were injected with the treatment. Thus, final group numbers included *N*=9 control-treated vertebrae and *N*=3 vertebrae per treatment dose. Postoperative analgesia consisted of phenylbutazone for 3 days postoperatively, with close indoor monitoring for 2 weeks postoperatively.

### Sheep experiment analyses

Analysis was performed using DXA, CT and microCT, FEA, histology and histomorphometry. DXA scans were performed pre- and post-induction (Konica Minolta mc5430DL). Next, a Philip's GEMINI TF Big Bore CT machine (0.8 mm thickness) was used to analyse bone formation postoperatively, monthly until being killed at 3 months. Although serial live animal imaging was performed, data evaluation was performed at the study end point (no interim evaluation of the results performed). Post-mortem, high-resolution microCT scanning of individual vertebrae was performed as per mouse studies. Cortical and trabecular analyses were performed on post-mortem individual sheep vertebrae. Cortical measurements were obtained per vertebral body either assessing the surface of injection or the opposing cortical surface. Serial measurements along the cortex were obtained (*N*=162 control-treated measurements and *N*=55 measurements per treatment dose). Trabecular measurements were made using an ROI excluding the cortical bone. FEA (biomechanical) was performed using microcT images converted to DICOM files using the SKyScan Dicom Converter software (DicomCT application, Skyscan 1172F, Skyscan). Tetrahedral 3D mesh models were created by drawing a rectangular VOI directly underneath the injection tract, using the ScanIP software (Simpleware Limited). FEAs were performed using the ABAQUS software (Dassault Systèmes) with boundary conditions set as encastre, constrained in all directions. Next, we applied a uniform compressive pressure of 0.5 MPa on the superior surface of the spine, to reproduce human intradiscal pressure experienced in relaxed standing. The von Mises stress experienced and total strain energy of the samples were analysed. All radiographic analyses, including ROI construction, were performed in a blinded manner. The significant and visually observed anabolic effect of rhNELL-1 in theory presented a potential for unblinding. For histology, tissues were formalin-fixed, resin-embedded and stained with haematoxylin and eosin, Goldner's Modified Trichrome and Von Kossa-MacNeal's Tetrachrome. Histological specimens were analysed using the Olympus BX51 microscopes and images acquired using MicroFire digital camera with the Picture Frame software (Optronics, Goleta, CA). Histomorphometry was performed as per mouse studies. Ob.N was assessed by × 400 images of Von Kossa–MacNeal's Tetrachrome (*N*=10, 9, 9 and 9 images per treatment group, respectively), while Oc.N was assessed by × 200 images of Goldner's Modified Trichrome staining (*N*=9, 10, 9 and 9 images per treatment group, respectively). Characteristic cell morphology with three or more nuclei was used to define Oc.N.

### Statistical analysis

Quantitative data are expressed at mean±s.e.m. unless otherwise described, with *P<0.05 and **P<0.01 considered significant. A Shapiro–Wilk test for normality was performed on all data sets. Homogeneity was confirmed by a comparison of variance test. Parametric data were analysed using an appropriate Student's *t*-test when two groups were being compared, or a one-way analysis of variance was used when more than two groups were compared, followed by a *post hoc* Tukey's test to compare the two groups. Nonparametric data were analysed with a Mann–Whitney *U*-test when two groups were being compared or a Kruskal–Wallis one-way analysis when more than two groups were compared. As appropriate, adjustments for multiple comparisons were performed using a Bonferroni correction (see Supplementary Tables 9 and 10). Sample size calculations were performed for experiments presented in Figs 5 and 6 and Supplementary Figs 6–8 as follows: for experiments presented in Fig. 5 and Supplementary Fig. 6 initial animal numbers were on the basis of an *α*=0.05, power=0.8 and an anticipated effect size of 3.73 (on the basis of our previously published data in sheep spinal fusion)^27^. For experiments presented in Fig. 6 and Supplementary Figs 7 and 8, initial animal numbers were on the basis of an *α*=0.05, power=0.8 and an anticipated effect size of 2.69 (on the basis of our previously published data in local rhNELL-1 injection in ovariectomized rats)^62^. No sample size calculations were performed for animal studies in Fig. 1, Supplementary Figs 1 and 2, as all available transgenic animals were examined.

## 

## Additional information

**How to cite this article:** James, A. W. *et al*. NELL-1 in the treatment of osteoporotic bone loss. *Nat. Commun*. 6:7362 doi: 10.1038/ncomms8362 (2015).

## Supplementary Material

Supplementary Information Supplementary Figures 1-8, Supplementary Tables 1-10, Supplementary Note 1 and Supplementary References
