# Author Correction: NELL-1 in the treatment of osteoporotic bone loss

**DOI:** 10.1038/s41467-021-20933-x

**Published:** 2021-01-13

**Authors:** Aaron W. James, Jia Shen, Xinli Zhang, Greg Asatrian, Raghav Goyal, Jin H. Kwak, Lin Jiang, Benjamin Bengs, Cymbeline T. Culiat, A. Simon Turner, Howard B. Seim, Benjamin M. Wu, Karen Lyons, John S. Adams, Kang Ting, Chia Soo

**Affiliations:** 1 Department of Orthopaedic Surgery and the Orthopaedic Hospital Research Center, UCLA and Orthopaedic Hospital, University of California, Los Angeles, California 90095 USA; 2 Division of Growth and Development, Section of Orthodontics, School of Dentistry, University of California, Los Angeles, California 90095 USA; 3 Department of Pathology and Laboratory Medicine, David Geffen School of Medicine, University of California, Los Angeles, California 90095 USA; 4 Department of Neurology, Easton Center for Alzheimer’s Disease Research, Molecular Biology Institute, University of California, Los Angeles, California 90095 USA; 5 Oak Ridge National Laboratory (ORNL), Oak Ridge, Tennessee 37830 USA; 6 Department of Veterinary Sciences, Colorado State University, Fort Collins, Colorado 80523 USA; 7 Department of Bioengineering and Department of Material Sciences, University of California, Los Angeles, California 90095 USA; 8 Division of Plastic and Reconstructive Surgery, Department of Surgery, David Geffen School of Medicine, University of California, Los Angeles, Los Angeles, California 90095 USA

Correction to: *Nature Communications* 10.1038/ncomms8362, published online 17 June 2015.

The original version of this Article contained an error in Supplementary Fig. 7, where the histology images in panel e were inadvertently duplicated from the images in panel g. The correct images have now been included in Fig. 7e in both the PDF and HTML versions of the Article.

## Supplementary information

Supplementary Materials

## Supplementary information

**Supplementary information** The online version contains supplementary material available at 10.1038/s41467-021-20933-x.

