# Supplementary material for: NELL-1 in the treatment of osteoporotic bone loss
Source: Nat Commun. 2015 Jun 17;6:7362. doi: 10.1038/ncomms8362 (PMC4557288; doi:10.1038/ncomms8362)
Supplement: Supplementary Information — Supplementary Figures 1-8, Supplementary Tables 1-10, Supplementary Note 1 and Supplementary References [file ncomms8362-s1.pdf]

Supplementary Materials

Supplementary Figures:

Supplementary Fig. 1.

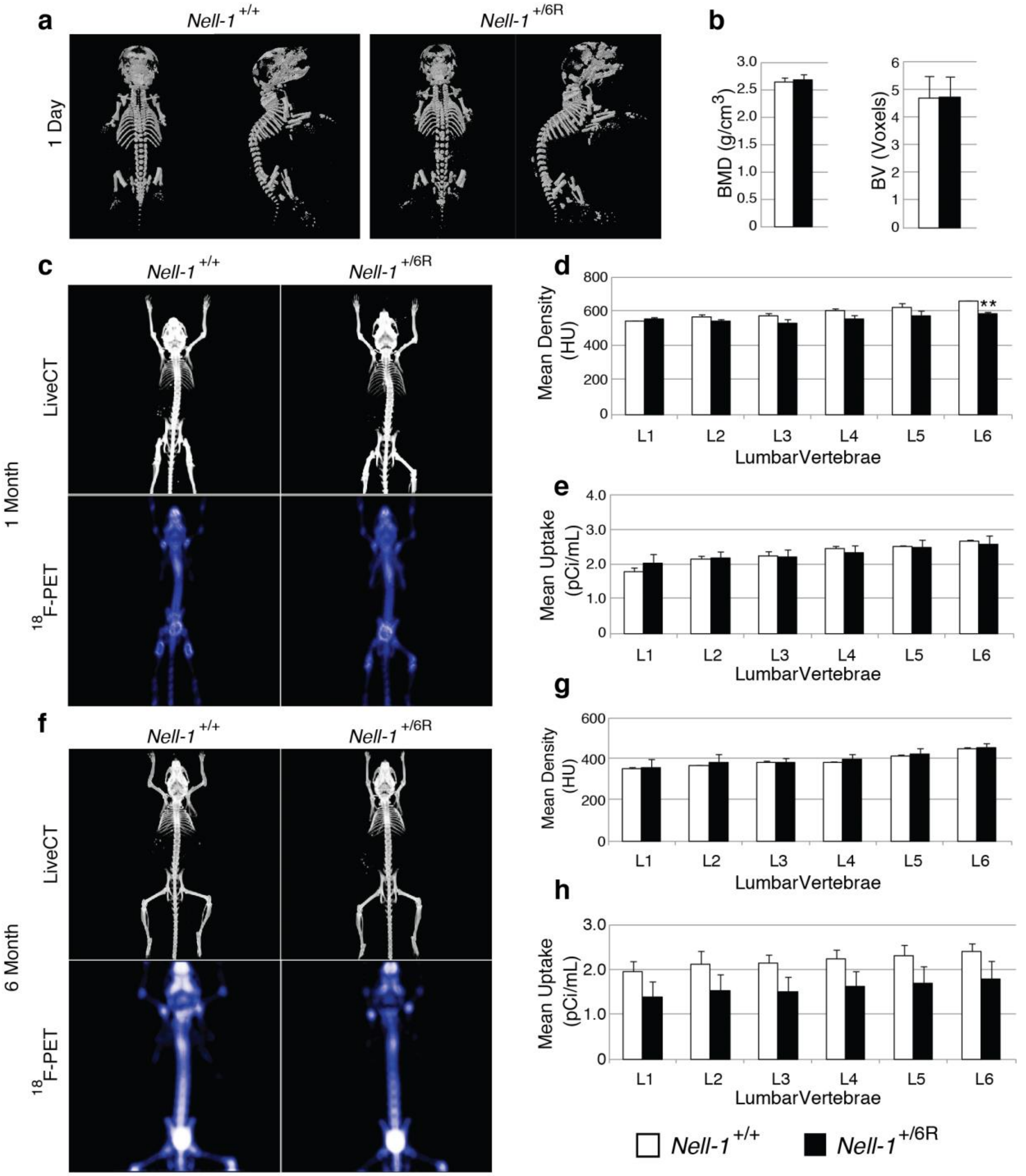

Supplementary Fig. 1. Skeletal Phenotype of Juvenile *Nell-1* Haploinsufficient Mice. **(a)** Representative post-mortem, 3D, micro Computed Tomography (microCT) reconstructions at birth of wildtype (*Nell-1*$^{+/+}$) and *Nell-1* haploinsufficient (*Nell-1*$^{+/6R}$) mice. The axial and appendicular skeletons were grossly identical. **(b)** Quantification of Bone Mineral Density (BMD) and Bone Volume (BV) of the entire lumbar spine at birth. N=10 mice per genotype. Histomorphometric analysis of newborn mice spines showed no statistical difference, presented in **Supplementary Table 1**. In addition, cellular proliferation and cell death showed no observed differences, as assessed by PCNA and TUNEL staining, respectively (*data not shown*). **(c)** Representative microCT and ^{18}F radioisotope incorporation images at 1 month of age. **(d)** BMD quantification at 1 month of age, stratified by lumbar vertebral level (L1-L6). N=8 mice per genotype. **(e)** ^{18}F-PET radioisotope quantification at 1 month, stratified by lumbar vertebral level (L1-L6). N=8 mice per genotype. **(f)** Representative microCT and ^{18}F-PET radioisotope incorporation images at 6 months of age. **(g,h)** Quantification of microCT and ^{18}F-PET, stratified by lumbar vertebral level (L1-L6). N=8 mice per genotype. Data points indicate means, while error bars represent one standard errors of the mean (SEM). *In vivo* experiments were performed without replicate, unless otherwise described. Parametric data was analyzed using an appropriate Student's *t*-test or a one-way ANOVA, followed by a post-hoc Tukey's test. Nonparametric data was analyzed with a Mann-Whitney U test or a Kruskal-Wallis one-way analysis. $^{**}P<0.01$ in comparison to *Nell-1*$^{+/+}$ values.

Supplementary Fig. 2.

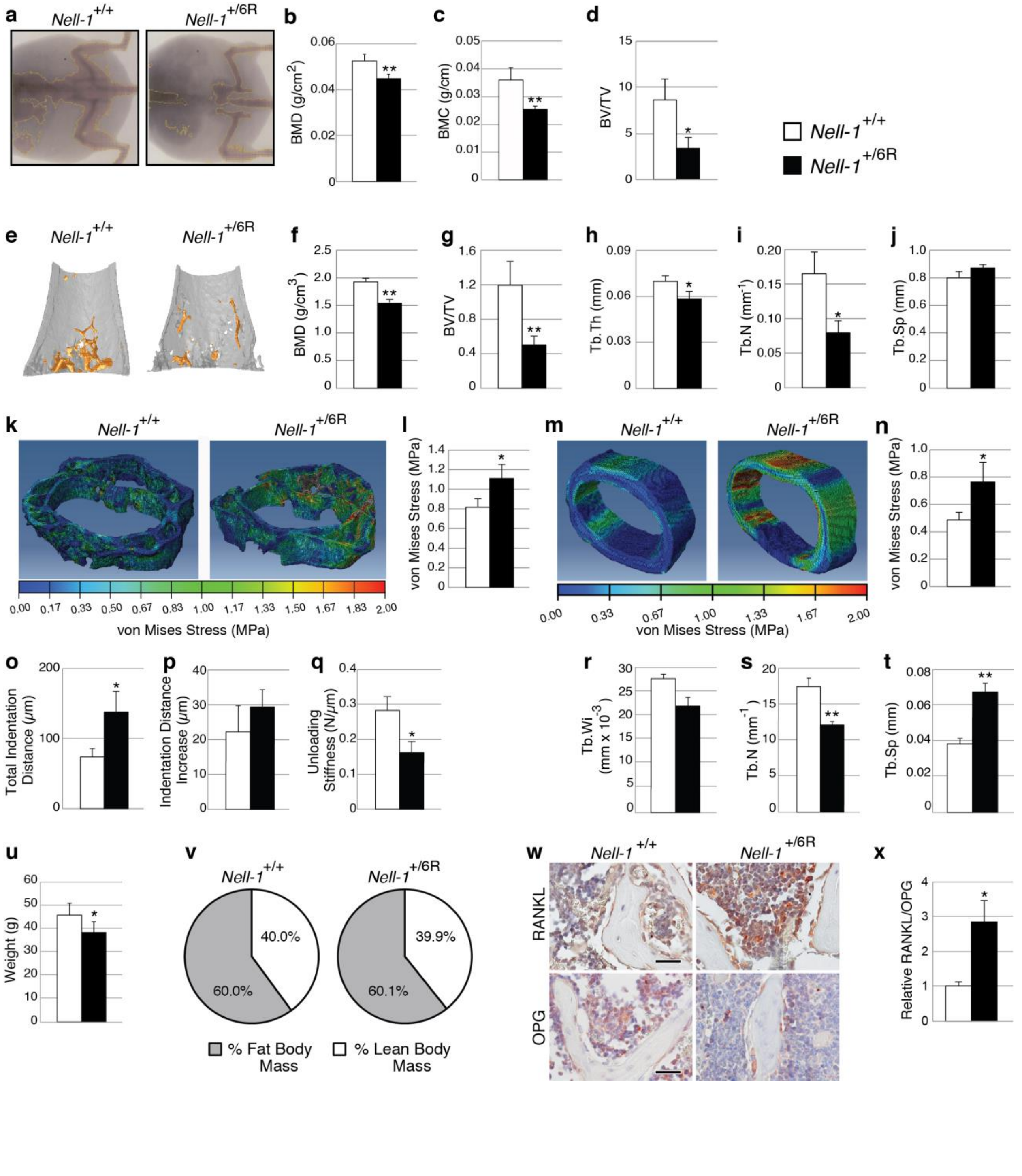

Supplementary Fig. 2. Skeletal Phenotype and Body Habitus of Aged *Nell-1* Haploinsufficient Mice. Mice were analyzed at 18 months of life. **(a)** Representative DXA images of wildtype (*Nell-1*$^{+/+}$) and *Nell-1*^{6R} heterozygote (*Nell-1*$^{+/6R}$) littermates. N=12 *Nell-1*$^{+/+}$ and 16 *Nell-1*$^{+/6R}$ mice were used for DXA analyses. **(b,c)** Mean Bone Mineral Density (BMD) and Bone Mineral Content (BMC) of the lumbar vertebrae among *Nell-1*$^{+/+}$ and *Nell-1*$^{+/6R}$ mice, assessed by DXA. (**d**) MicroCT quantification of BV/TV in the lumbar spine of aged *Nell-1*$^{+/+}$ and *Nell-1*$^{+/6R}$ mice. N=12 *Nell-1*$^{+/+}$ and 19 *Nell-1*$^{+/6R}$ individual vertebrae per genotype. **(e)** MicroCT reconstructions of the distal femur of aged *Nell-1*$^{+/+}$ and *Nell-1*$^{+/6R}$ mice. **(f-j)** Trabecular microCT quantifications of the distal femur, including **(f)** BMD, **(g)** BV/TV, **(h)** Tb.Th, **(i)** Tb.N, and **(j)** Tb.Sp. N=10 individual vertebrae per genotype. **(k,l)** Finite element analysis (FEA) and quantification of von Mises stress of aged *Nell-1*$^{+/+}$ and *Nell-1*$^{+/6R}$ lumbar spines. N=6 *Nell-1*$^{+/+}$ and 8 *Nell-1*$^{+/6R}$ mice. **(m,n)** Finite element analysis (FEA) and quantification of von Mises stress of aged *Nell-1*$^{+/+}$ and *Nell-1*$^{+/6R}$ distal femurs. Red color indicates areas of high stress. N=6 *Nell-1*$^{+/+}$ and 8 *Nell-1*$^{+/6R}$ mice. **(o-q)** BioDent™ Mechanical Testing in aged *Nell-1* haploinsufficient mice. Mechanical properties were examined between the sacral (S1) vertebral body of *Nell-1*$^{+/+}$ and *Nell-1*$^{+/6R}$ aged littermates. N=3 mice per genotype, with N=6 measurements per mouse performed. **(o)** Total Indentation Distance, calculated by measuring the maximum indentation distance achieved during a measurement. **(p)** Indentation Distance Increase, calculated by measuring the difference between the depths reached at peak force during the first indentation cycle and last indentation cycle. **(q)** Unloading Stiffness, as calculated by evaluating the top portion of the unloading section

of the force displacement curve. **(r-t)** Trabecular analyses of histological sections of lumbar vertebrae of aged *Nell-1*$^{+/+}$ and *Nell-1*$^{+/6R}$ mice, including **(r)** Trabecular Width (Tb.Wi), **(s)** Number (Tb.N), and **(t)** Spacing (Tb.Sp). **(u)** Mean weight among *Nell-1*$^{+/+}$ and *Nell-1*$^{+/6R}$ mice, showing a significant reduction in overall body weight among *Nell-1*$^{+/6R}$ mice. N=12 *Nell-1*$^{+/+}$ and 16 *Nell-1*$^{+/6R}$ mice. **(v)** Nuclear Magnetic Resonance (NMR) analysis of % fat body mass and % lean body mass, showing no significant difference between *Nell-1*$^{+/+}$ and *Nell-1*$^{+/6R}$ mice. N=12 *Nell-1*$^{+/+}$ and 16 *Nell-1*$^{+/6R}$ mice were used for NMR analyses. **(w,x)** RANKL and OPG immunohistochemistry in aged *Nell-1*$^{+/+}$ and *Nell-1*$^{+/6R}$ mouse spines. Semi-quantification is expressed as relative RANKL^{+} to OPG^{+} bone lining cells. Black scale bar: 25 um. Data points indicate means, while error bars represent one SEM. *In vivo* experiments were performed without replicate, unless otherwise described. Parametric data was analyzed using an appropriate Student's *t*-test or a one-way ANOVA, followed by a post-hoc Tukey's test. Nonparametric data was analyzed with a Mann-Whitney U test or a Kruskal-Wallis one-way analysis. $*P<0.05$, $**P<0.01$ in comparison to *Nell-1*$^{+/+}$ values.

Supplementary Fig. 3.

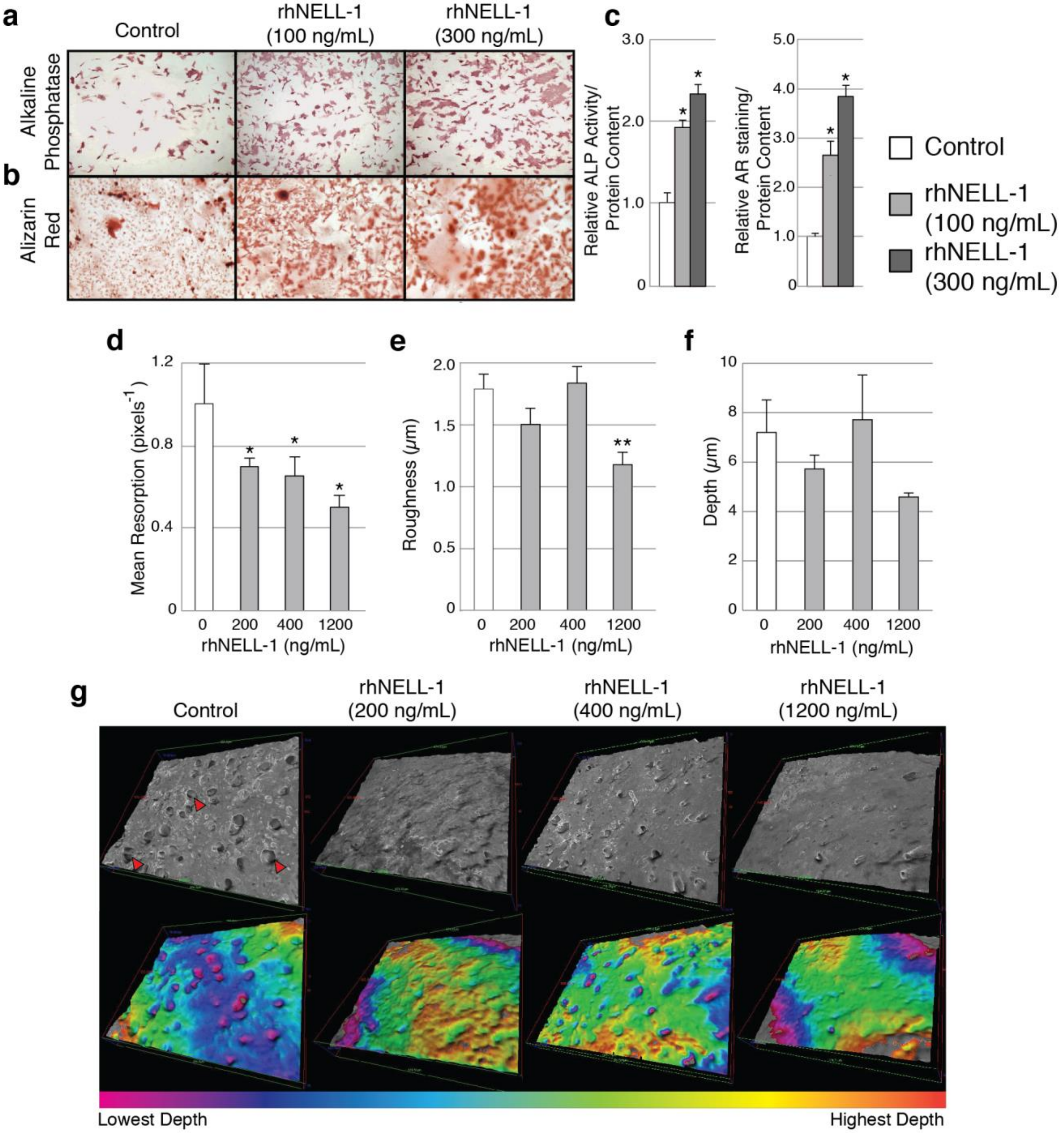

Supplementary Fig. 3. RhNELL-1 *In Vitro* Effects in OB precursors and OC precursors. Cells were derived from the marrow of wildtype mice for either OB precursor or OC precursor assays. **(a-c)** OB precursor differentiation assays. **(a)** Alkaline Phosphatase (ALP) staining among wildtype OB precursors with or without rhNELL-1 (100 & 300 ng/mL) at 7 days of osteogenic differentiation. **(b)** Alizarin Red (AR) staining of bone nodule formation at 14 days of osteogenic differentiation with or without rhNELL-1 (100 & 300 ng/mL). **(c)** Photometric quantification of ALP activity and AR staining, normalized to total protein content (N=4 wells per group). **(d-g)** Calvarial disc bone resorption assays among wildtype OC precursors treated with or without rhNELL-1 (0-1200 ng/mL), after 5 days resorption (N=4 resorption discs per group). **(d)** Photographic quantification of bone resorption as determined by Toluidine Blue staining. **(e,f)** Quantification of calvarial resorption as assessed by SEM of **(e)** average surface roughness (N=12 measurements per group) and **(f)** average pit depth (N=30 measurements per group). **(g)** Reconstructions of calvarial disc resorption assays based on scanning electron microscopy (SEM). Red arrows indicate representative resorption pits. Colorized SEM highlights resorption pits in purple. Data points indicate means, while error bars represent one SEM. *In vitro* experiments were performed in biological triplicate, unless otherwise described. Parametric data was analyzed using an appropriate Student's *t*-test or a one-way ANOVA, followed by a post-hoc Tukey's test. Nonparametric data was analyzed with a Mann-Whitney U test or a Kruskal-Wallis one-way analysis. $^*P<0.05$, $^{**}P<0.01$.

Supplementary Fig. 4.

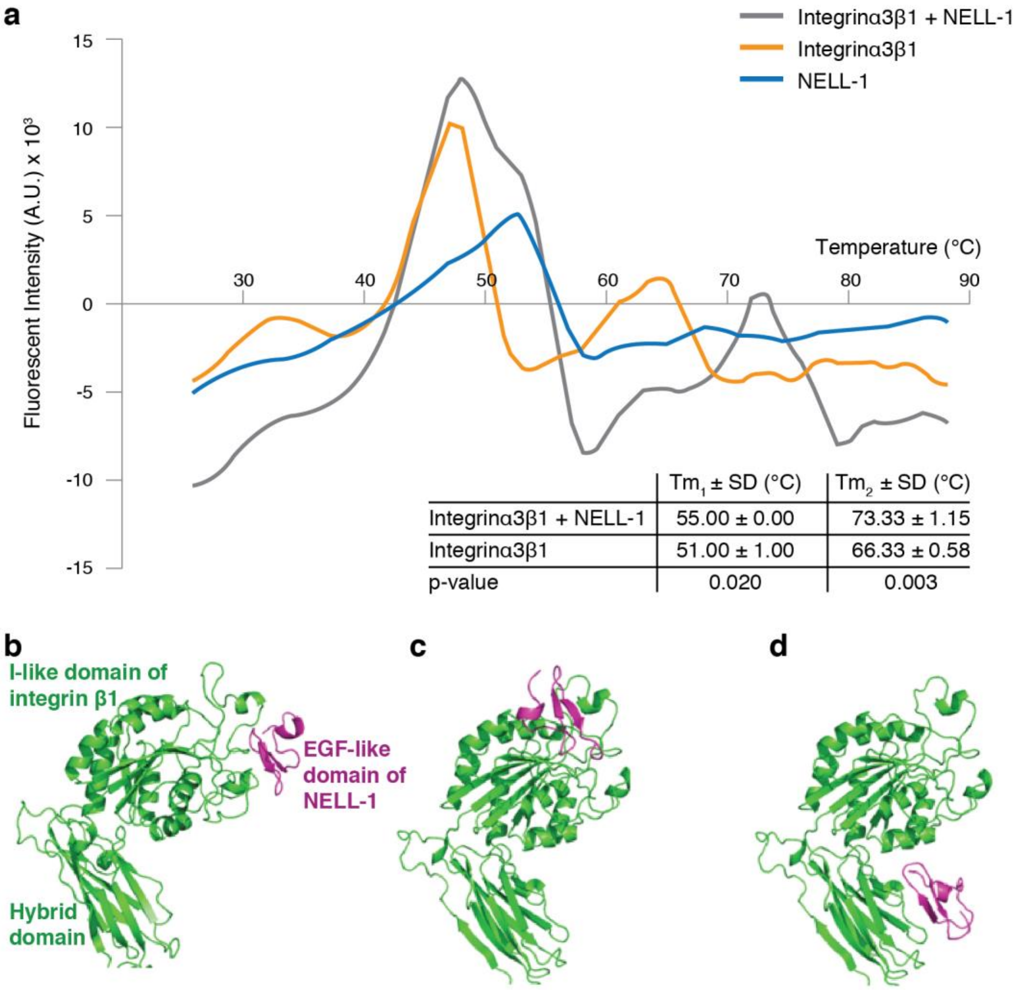

	Tm_1 ± SD (°C)	Tm_2 ± SD (°C)
Integrinα3β1 + NELL-1	55.00 ± 0.00	73.33 ± 1.15
Integrinα3β1	51.00 ± 1.00	66.33 ± 0.58
p-value	0.020	0.003

Supplementary Fig. 4. Evidence of NELL-1 - Integrinβ1 binding. Please note that evidence for NELL-1 binding to Integrinβ1 by co-immunoprecipitation assays has been previously published[1]. **(a**) Thermal shift assays of NELL-1 and integrin α3β1, shown as the first derivative. The integrin α3β1 heterodimer was used so as to maintain protein bioactivity (R&D Systems, 2840-A3-050). Two Tm from integrin α3β1 were shifted upon addition of NELL-1. Assays were performed in triplicate with a representative assay shown, with means and standard deviations of Tm shown in the chart below. **(b-d)** Docking stimulation of NELL-1 interaction. The three top-scoring models of NELL-1 - integrin β1 interaction predicted by docking simulation by using RosettaDock are shown. The structure of integrin β1 was taken from the crystal structure of α5β1 integrin headpiece (PDB entry 3VI3). The I-like domain and hybrid domain of integrin β1 (in a green color) are highlighted. The model of the EGF-like domain of NELL-1 was built by using Robetta protein structure prediction server (amino acid 549-582 of NELL-1 protein). **(b)** The model predicts that the EGF-like domain of NELL-1 binds to the I-like domain of the integrin β1, the same binding pocket as previously reported[2]. Its predicted total energy is -230kcal/mol. **(c)** The model predicts that the EGF-like domain of NELL-1 binds to the I-like domain of the integrin β1 at an alternative binding pocket. Its predicted total energy is -235kcal/mol. **(d)** The model predicts that the EGF-like domain of NELL-1 binds to the hybrid domain of the integrin β1. Its predicted total energy is -231kcal/mol. *In vitro* experiments were performed in biological triplicate, unless otherwise described.

Supplementary Fig. 5.

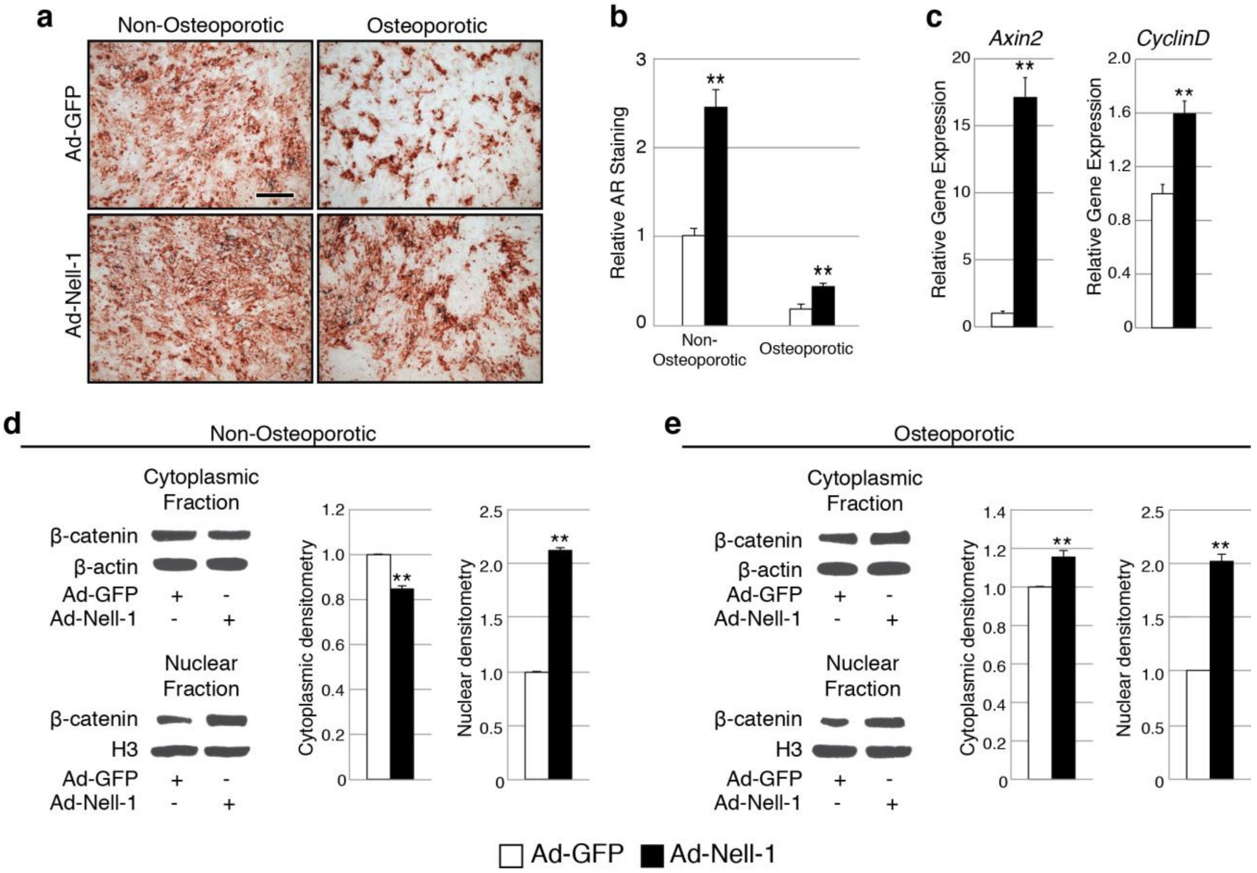

Supplementary Fig. 5. Nell-1 signaling increases Wnt/β-catenin activity in human BMSCs derived from osteoporotic and non-osteoporotic patients. See **Supplementary Table 2** for details of patient samples. (**a**) Alizarin red staining among osteoporotic and non-osteoporotic hBMSCs, assessed at 11 days. (**b)** Photographic quantification of Alizarin red staining. (**c**) *Axin2* and *CyclinD* mRNA expression 3 days after viral infection in non-osteoporotic hBMSCs. (**d,e**) Western blot of β-catenin of both cytoplasmic and nuclear fractions of hBMSCs overexpressing *Nell-1*. A GFP encoding adenovirus (ad-GFP) was used as control. **(d)** Western blot and quantification from non-osteoporotic hBMSCs. **(e)** Western blot and quantification from osteoporotic hBMSCs. Black scale bar: 100 μm. All experiments using human BMSC were performed in triplicate wells. Data points indicate means, while error bars represent one SEM. *In vitro* experiments were performed in biological triplicate, unless otherwise described. Parametric data was analyzed using an appropriate Student's *t*-test or a one-way ANOVA, followed by a post-hoc Tukey's test. Nonparametric data was analyzed with a Mann-Whitney U test or a Kruskal-Wallis one-way analysis. $^{**}P<0.01$. Confirming efficacy of transection, Ad-Nell-1 resulted in a 17.5 fold increase in *Nell-1* mRNA transcripts (*data not shown*).

Supplementary Fig. 6.

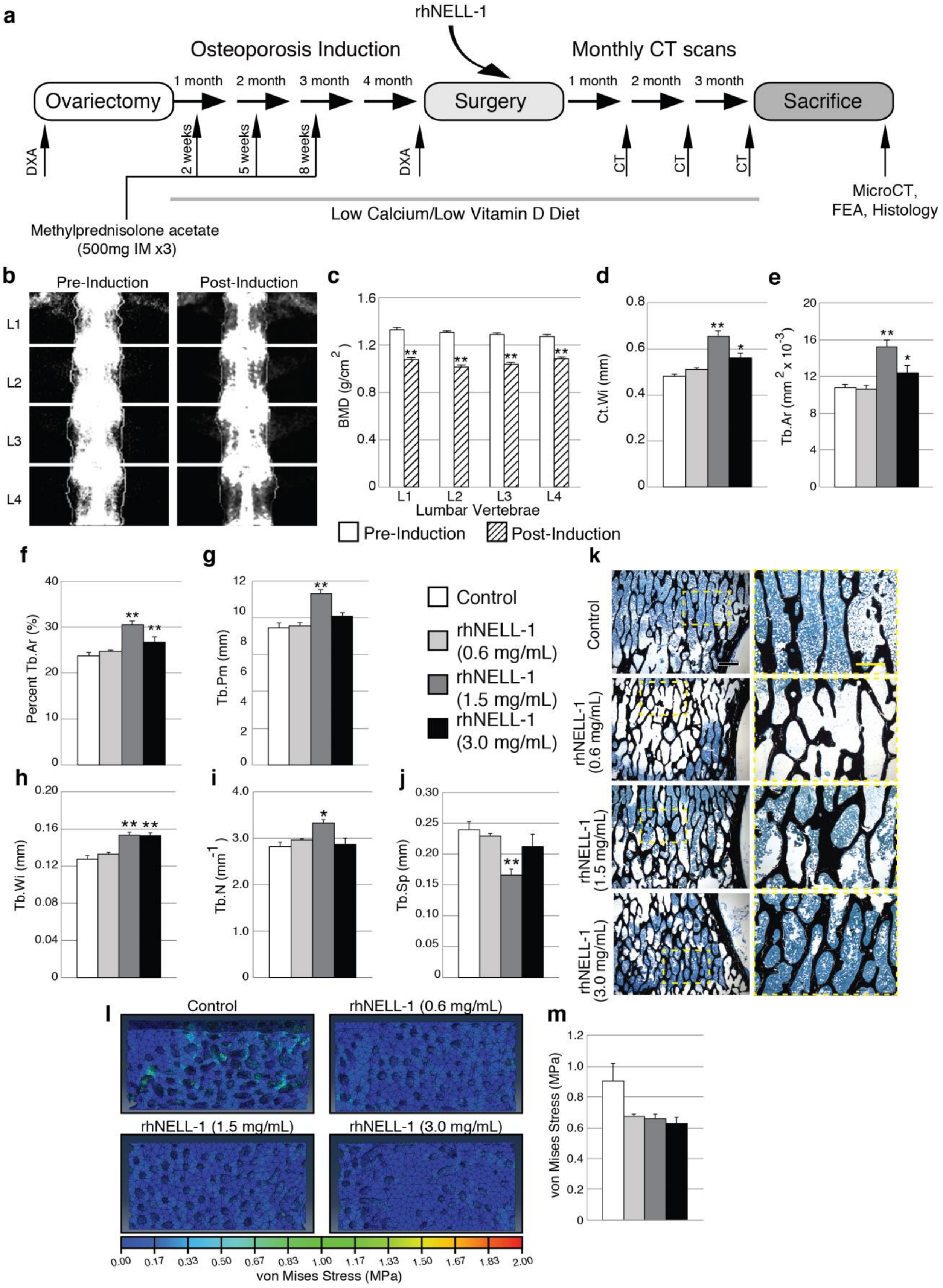
a
rhNELL-1
Osteoporosis Induction
Monthly CT scans
Ovariectomy
1 month
2 month
3 month
4 month
Surgery
Sacrifice
DXA
2 weeks
5 weeks
8 weeks
CT
MicroCT, FEA, Histology
Low Calcium/Low Vitamin D Diet
Methylprednisolone acetate (500mg IM x3)
b
Pre-Induction
Post-Induction
L1
L2
L3
L4
c
BMD (g/cm2)
Lumbar Vertebrae
Pre-Induction
Post-Induction
d
Ct.Wi (mm)
e
Tb.Ar (mm2 x 10-3)
f
Percent Tb.Ar (%)
g
Tb.Pm (mm)
Control
rhNELL-1 (0.6 mg/mL)
rhNELL-1 (1.5 mg/mL)
rhNELL-1 (3.0 mg/mL)
h
Tb.Wi (mm)
i
Tb.N (mm-1)
j
Tb.Sp (mm)
k
l
Control
rhNELL-1 (0.6 mg/mL)
rhNELL-1 (1.5 mg/mL)
rhNELL-1 (3.0 mg/mL)
von Mises Stress (MPa)
m
von Mises Stress (MPa)

Supplementary Fig. 6. Sheep Study Design, Induction of Osteoporosis and Additional Data. **(a)** Study design. Osteoporosis was induced over four months time by a combination of ovariectomy, steroid administration, and dietary deficiency in Vitamin D and Calcium. After successful induction of osteoporosis, rhNELL-1 was applied by intravertebral body injection. Postoperative analysis included monthly CT scans. Harvest at 3 months post-operative was followed by high-resolution microCT, FEA, and histological analysis. **(b)** Representative DXA images before and after osteoporotic induction. **(c)** Quantification of Bone Mineral Density (BMD) as determined by DXA pre- and post-osteoporotic induction, stratified by lumbar (L) vertebral level. (**d-k**) RhNELL-1 Protein Intravertebral Injection Increases Bone Formation in Osteoporotic Sheep by Histology. Focus of study included the side of the vertebral body most distant from rhNELL-1 injection. N=9 random images per treatment group. **(d-j)** Histomorphometric quantification of cortical and trabecular measurements, including **(d)** Cortical Width (Ct.Wi), **(e)** Trabecular bone Area (Tb.Ar), **(f)** Percentage Trabecular bone Area (% Tb.Ar), **(g)** Trabecular bone Perimeter (Tb.Pm), **(h)** Trabecular bone Width (Tb.Wi), **(i)** Trabecular bone Number (Tb.N) and **(j)** Trabecular bone Spacing (Tb.Sp). **(k)** Representative images of Von Kossa MacNeal's Tetrachrome (VKMT) staining of the area contralateral to the injection site. **(l)** Images and **(m)** quantification of biomechanical stress, determined by Finite Element Analysis (FEA). The region of interest is a rectangle adjacent to the injection tract. Black scale bar: 1mm; Yellow scale bar: 0.5 mm. N=9 control-treated vertebrae, N=3 vertebrae per treatment dose. Data points indicate means, while error bars represent one SEM. *In vivo* experiments were performed without replicate, unless otherwise described. Parametric data was analyzed using an appropriate Student's *t*-test or a one-way ANOVA, followed by a post-hoc

Tukey's test. Nonparametric data was analyzed with a Mann-Whitney U test or a Kruskal-Wallis one-way analysis. *P<0.05, **P<0.01 in comparison to control.

Supplementary Fig. 7.

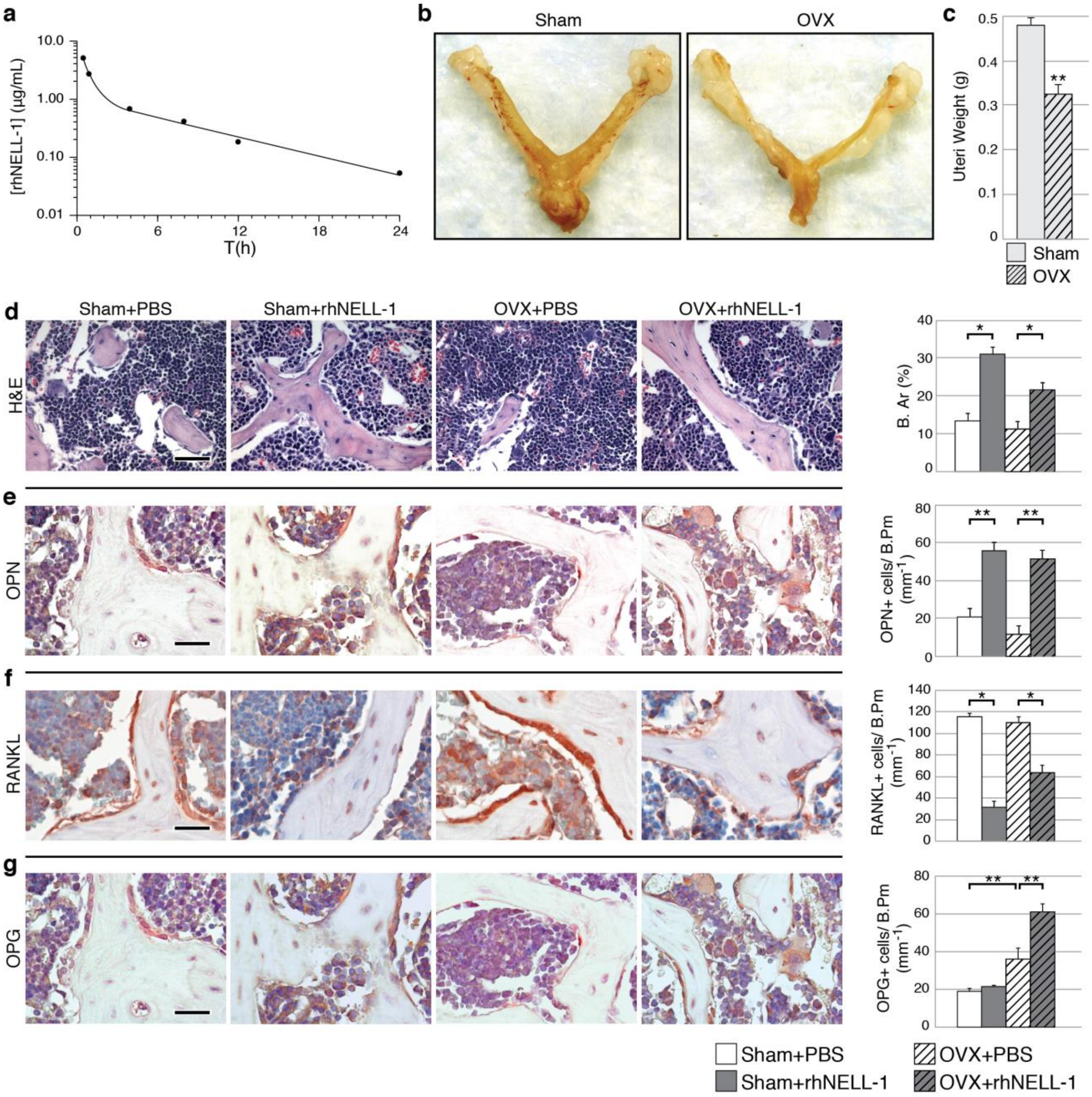

Supplementary Fig. 7. Systemic NELL-1 treatment and Additional Data. (**a**) Serum concentration of rhNELL-1 after a single intravenous injection. N=3 samples per timepoint. **(b,c)** Induction of osteoporosis was performed by ovariectomy (OVX) and ensuing bone loss over a five week period. OVX was confirmed by post-mortem examination of uterine atrophy and mean weight (N=13 and 15 mice, respectively). (**d-**) RhNELL-1 was next administered by tail vein injection (1.25 mg/kg q48hr), sacrificed after four weeks. **(d)** H&E staining and quantification of percentage Bone Area (B. Ar). **(e)** Osteopontin (OPN) immunohistochemical staining, and quantification of OPN^+ bone-lining cells per B.Pm. **(f)** RANKL immunohistochemical staining and quantification of $RANKL^+$ bone lining cells per B.Pm. **(g)** OPG immunohistochemical staining and quantification of OPG^+ bone lining cells per B.Pm (N=18-30 images per group for each immunohistochemical quantification). Data points indicate means, while error bars represent one SEM. *In vivo* experiments were performed without replicate, unless otherwise described. Parametric data was analyzed using an appropriate Student's *t*-test or a one-way ANOVA, followed by a post-hoc Tukey's test. Nonparametric data was analyzed with a Mann-Whitney U test or a Kruskal-Wallis one-way analysis. *P<0.05, **P<0.01 in comparison to PBS control.

Supplementary Fig. 8.

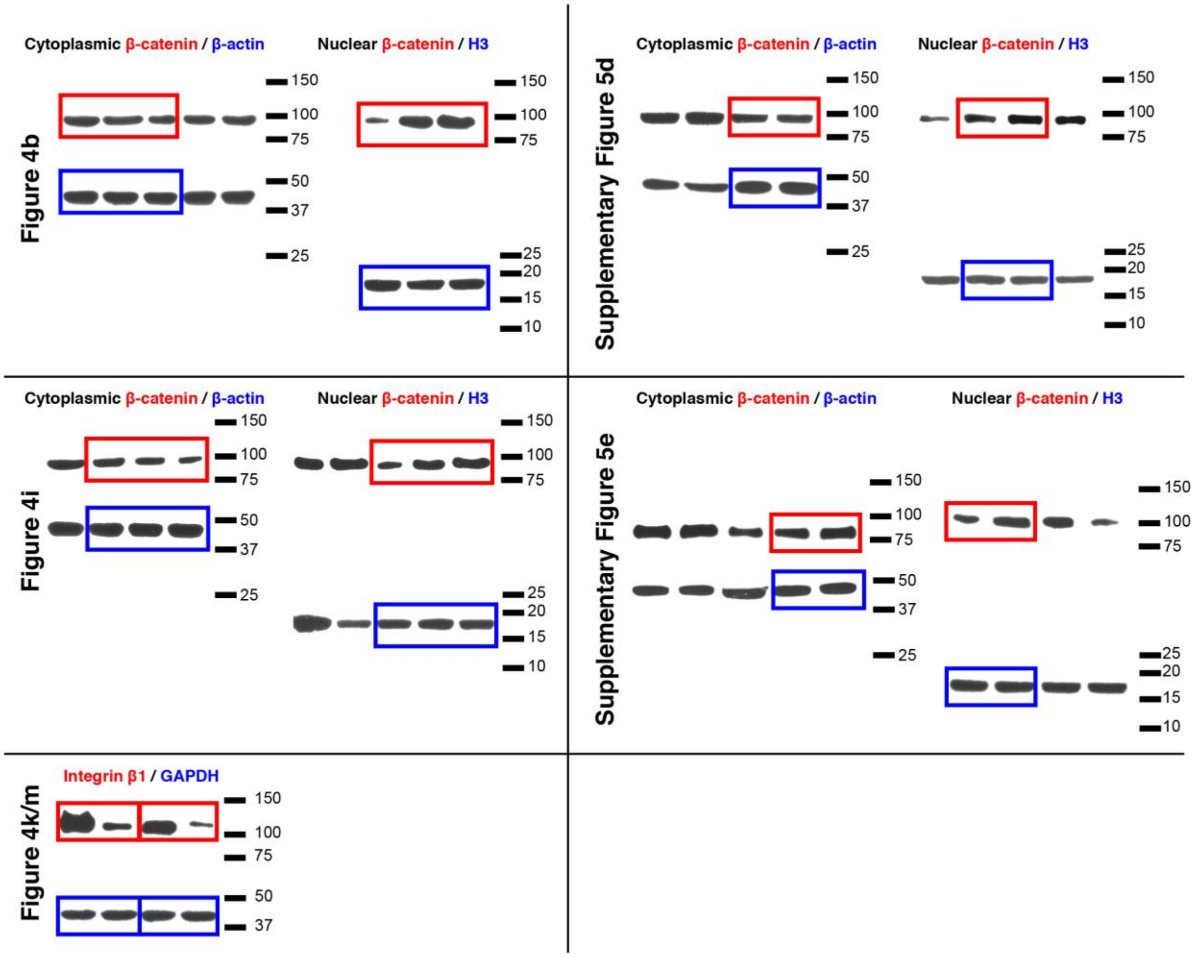

Supplementary Fig. 8. Supplementary western blots. Red and blue rectangles indicate proteins of interest and controls, respectively.

Supplementary Tables:

Supplementary Table 1. Spine histomorphometric measurements at birth with *Nell-1* haploinsufficiency.

	Nell-1$^{+/+}$ (Mean±SEM)	*Nell-1*$^{+/6R}$ (Mean±SEM)	*p-value*
Percent Tb.Ar (%)	21.71 ± 2.16	21.07 ± 0.814	0.405
Tb.Wi (μm)	1.321 ± 0.208	0.963 ± 0.114	0.084
Tb.N (μm^{-1})	0.252 ± 0.092	0.276 ± 0.030	0.495
Tb.Sp (μm)	4.38 ± 0.930	3.01 ± 0.335	0.065

Material is based on histomorphometric analysis of N=5 samples per genotype. Data is presented as mean ± one standard error of the mean (SEM).

Supplementary Table 2. Demographics of samples for human BMSC derivation. AVN: Avascular necrosis.

Patient #	Age	Gender	H/o Osteoporosis	Site	Indication
1	23	F	N	Femur	Fracture
2	47	M	N	Femur	AVN
3	54	F	N	Femur	Joint replacement
4	47	F	N	Femur	Joint replacement
5	32	F	N	Femur	Joint replacement
6	83	F	Y	Femur	Fracture
7	91	F	Y	Femur	Fracture
8	71	F	Y	Femur	Fracture

Supplementary Table 3. Treatment composition for sheep intervertebral body injections.

	Control	rhNELL-1 (low)	rhNELL-1 (mid)	rhNELL-1 (high)
Volume Hyaluronic Acid	500 μL	500 μL	500 μL	500 μL
Mass β-TCP	50 mg	50 mg	50 mg	50 mg

Concentration rhNELL-1	0 mg/ml	0.6 mg/ml	1.5 mg/ml	3.0 mg/ml
Total Dose rhNELL-1	0 mg	0.3 mg	0.75 mg	1.5 mg
Vertebral body N	9	3	3	3

Supplementary Table 4. Histomorphometric measurements after sheep intervertebral body injections. Data is presented as mean ± one standard error of the mean (SEM).

		Control	rhNELL-1 (low)	rhNELL-1 (mid)	rhNELL-1 (high)
Contra. Ct.Wi	Mean±SEM	0.645±0.012	0.751±0.028	0.747±0.036	0.749±0.032
	p-value		*0.020*	*0.028*	*0.023*
Ipsi. Ct.Wi	Mean±SEM	0.660±0.012	0.819±0.028	0.830±0.032	0.778±0.028
	p-value		*< 0.001*	*< 0.001*	*0.002*
Tb.Th	Mean±SEM	0.238±0.002	0.263±0.002	0.259±0.003	0.263±0.001
	p-value		*0.002*	*0.007*	*0.002*
Tb.N	Mean±SEM	0.973±0.015	1.267±0.253	1.032±0.050	1.006±0.039
	p-value		*< 0.001*	*0.529*	*0.859*
Tb.Sp	Mean±SEM	0.615±0.006	0.563±0.001	0.582±0.003	0.577±0.004
	p-value		*0.002*	*0.022*	*0.011*

.

Supplementary Table 5. Pharmacokinetics of intravenous rhNELL-1 in mice.

	A (μg/mL)	α	$T_{1/2\alpha}$ (h)	B (μg/mL)	β	$T_{1/2\beta}$ (h)	AUC (μg/mL)	Cmax calc (μg/mL)
FITC-rhNELL-1	8.620	1.602	0.433	1.000	0.126	5.520	13.346	9.620

Supplementary Table 6. Antibodies used.

Antibody	Vendor	Cat. No.	Concentration	References	Validation Profile URL
Anti-Axin2	Abcam	AB32197	1 µg/ml	PMID: 22928951	http://1degreebio.org/reagents/product/104112/?qid=749839
Anti-β-actin	Santa Cruz	sc-1616	200 ng/ml	PMID: 23283970	http://1degreebio.org/reagents/product/1309/?qid=749854
Anti-β-catenin, active	Millipore	05665	1 µg/ml		http://1degreebio.org/reagents/product/869393/?qid=750604
Anti-β-catenin	BD Biosciences	610153	250 ng/ml	PMID: 17438527	http://1degreebio.org/reagents/product/862646/?qid=750591
Anti-GAPDH	Genetex, Inc	GTX100118	50 ng/ml	PMID: 25178567	http://1degreebio.org/reagents/product/1902584/?qid=830656
Anti-H3	Abcam	AB8898	1 µg/ml	PMID: 24169366	http://1degreebio.org/search/?search=AB8898&in=antibodies
Anti-Nell-1	Genetex, Inc.	GTX 111493	5 µg/ml	PMID: 25376942	http://1degreebio.org/reagents/product/1934501/?qid=750581
Anti-Nell-1	Allele Biotechnology	PAB-11648	5 µg/ml	PMID: 25376942	
Anti-PCNA	Dako	M0879	1:10,000 dilution	PMID: 1981239	
Anti-RANKL	Novus Biologicals	NB100-80849	5 µg/ml		http://www.antibodypedia.com/gene/3366/TNFSF11/antibody/71905/NB100-80849
Anti-Integrinb1	Abcam	AB95623	2 µg/ml	PMID: 10194438	http://1degreebio.org/reagents/product/327726/?qid=750547
Anti-OPG	Abcam	AB73400	5 µg/ml		http://1degreebio.org/reagents/product/333174/?qid=749915
Anti-Osteocalcin	Santa Cruz	Sc-30044	2 µg/ml	PMID: 22238668	
Anti-Osteopontin	Abcam	AB8448	1:200 dilution	PMID: 24586387	http://1degreebio.org/reagents/product/1162507/?qid=749910

Supplementary Table 7. List of ELISA assays used.

**Protein**	**Cat. No.**	**Manufacturer**
PINP (Procollagen I N-terminal Propeptide)	AC-33F1	ImmunoDiagnostic Systems, Inc
TRAP (Tartrate-Resistant Acid Phosphatase)-5b	SB-TR103	ImmunoDiagnostic Systems, Inc
CTX (C-Terminal Telopeptide)	AC-07F1	ImmunoDiagnostic Systems, Inc

Supplementary Table 8. List of primers and sequences used.

**Gene**	**Species**	**Forward Sequence**	**Reverse Sequence**
Alp	Mouse	TGCCACTGTGAGAAGACCTG	TGCACAGGAAGTGAGTCTGG
Axin2	Mouse	TCCAGTCCACCAAACCTATGC	CTGGTCAAAGGGGAATCGGG
Bmp2	Mouse	TCTTCCGGGAACAGATACAGG	TGGTGTCCAATAGTCTGGTCA
Bmp4	Mouse	TTCCTGGTAACCGAATGCTGA	CCTGAATCTCGGCGACTTTTT
Bmp7	Mouse	ACGGACAGGGCTTCTCCTAC	ATGGTGGTATCGAGGGTGGAA
C-Myc	Mouse	TCTCCATCCTATGTTGCGGTC	TCCAAGTAACTCGGTCATCATCT
CyclinD	Mouse	CTGGAAGAAGTCTGCGTCGG	GTCTTGCCAAAGCGGTTCAG
Gapdh	Mouse	TGCACCACCAACTGCTTAGC	CCACCACCCTGTTGCTGTAG
Nell-1	Mouse	TCCTGGGTAGATGGTGACAA	CATTGGCCAGAAATATGCAC
Ocn	Mouse	GCAATAAGGTAGTGAACAGACTCC	AGCAGGGTTAAGCTCACACTG
Opn	Mouse	ATCTCACCATTCGGATGAGTCT	TCAGTCCATAAGCCAAGCTATCA
Runx2	Mouse	CGGTGCAAACTTTCTCCAGGA	GCACTCACTGACTCGGTTGG
Axin2	Human	CTCCCCACCTTGAATGAAGA	ACTGGGTCGCTTCTCTTGAA
CyclinD	Human	ATGGAGGGCGGATTGGAAATGA	TCGGTGTCCTACTTCAAAGTGTG
Gapdh	Human	ATGGGGAAGGTGAAGGTCG	GGGGTCATTGATGGCAACAATA

Supplementary Table 9. Results of 5 day intravenous toxicity testing of rhNELL-1, including mean animal and organ body weights.

		0 mg/kg/day	1.25 mg/kg/day	2.5 mg/kg/day	6.25 mg/kg/day
**Body Weight (g)**	Mean ± SEM	30.75 ± 0.48	31.25 ± 0.48	31.25 ± 1.03	30.75 ± 1.03
	p-value		*0.244*	*0.341*	*0.500*
**Heart Weight (g)**	Mean ± SEM	0.161 ± 0.009	0.162 ± 0.002	0.173 ± 0.01	0.157 ± 0.005
	p-value		*0.454*	*0.190*	*0.374*
**Lungs Weight (g)**	Mean ± SEM	0.211 ± 0.01	0.21 ± 0.008	0.233 ± 0.019	0.218 ± 0.015
	p-value		*0.447*	*0.188*	*0.369*
**Thymus Weight (g)**	Mean ± SEM	0.078 ± 0.003	0.077 ± 0.013	0.087 ± 0.004	0.069 ± 0.004
	p-value		*0.464*	*0.073*	*0.060*
**Stomach Weight (g)**	Mean ± SEM	0.426 ± 0.02	0.402 ± 0.022	0.329 ± 0.017	0.346 ± 0.019
	p-value		*0.226*	*0.006*	*0.014*
**Duodenum Weight (g)**	Mean ± SEM	0.134 ± 0.026	0.137 ± 0.026	0.115 ± 0.022	0.15 ± 0.019
	p-value		*0.474*	*0.292*	*0.326*
**Liver Weight (g)**	Mean ± SEM	1.987 ± 0.121	2.109 ± 0.041	2.022 ± 0.126	1.937 ± 0.047
	p-value		*0.199*	*0.426*	*0.359*
**Spleen Weight (g)**	Mean ± SEM	0.119 ± 0.005	0.147 ± 0.029	0.128 ± 0.009	0.13 ± 0.014
	p-value		*0.204*	*0.226*	*0.241*
**Kidneys Weight (g)**	Mean ± SEM	0.479 ± 0.017	0.530 ± 0.020	0.5242 ± 0.027	0.533 ± 0.017
	p-value		*0.046*	*0.102*	*0.081*
**Brain Weight (g)**	Mean ± SEM	0.479 ± 0.010	0.477 ± 0.023	0.478 ± 0.009	0.446 ± 0.011
	p-value		*0.462*	*0.457*	*0.076*
**Testes Weight (g)**	Mean ± SEM	0.218 ± 0.012	0.213 ± 0.011	0.213 ± 0.021	0.204 ± 0.09
	p-value		*0.386*	*0.414*	*0.187*

**Femur Weight (g)**	Mean ± SEM<br>*p-value*	0.069 ± 0.002	0.093 ± 0.006<br>*0.008*	0.094 ± 0.01<br>*0.052*	0.087 ± 0.004<br>*0.006*

N=4 animals per treatment group. No statistically significant differences were found with rhNELL-1 treatment, although a non-significant trend toward reduced stomach weight and increased bone weight was observed. Data is presented as mean ± standard error of the mean (SEM). *P<0.0014 in comparison to 0 mg/kg/day group.

Supplementary Table 10. Results of 5 day intravenous toxicity testing of rhNELL-1, including hematology and chemistry panels.

		**0 mg/kg/day**	**1.25 mg/kg/day**	**2.5 mg/kg/day**	**6.25 mg/kg/day**
**WBC ($x10^3$)**	Mean ± SEM<br>*p-value*	4.1 ± 0.85	*4.6* ± 0.95<br>*0.370*	*4.9* ± 0.7<br>*0.257*	*4.3* ± 0.35<br>*0.450*
**RBC ($x10^6$)**	Mean ± SEM<br>*p-value*	8.7 ± 0.25	*8.8* ± 0.2<br>*0.437*	*8.6* ± 0.25<br>*0.401*	*8.8* ± 0.35<br>*0.455*
**Hgb (gm/dl)**	Mean ± SEM<br>*p-value*	13.6 ± 0.3	*14.1* ± 0.3<br>*0.139*	*14.4* ± 0.4<br>*0.082*	*14.6* ± 0.5<br>*0.167*
**HCT (%)**	Mean ± SEM<br>*p-value*	49.7 ± 1.0	50.1 ± 1.15<br>*0.401*	51.1 ± 1.6<br>*0.256*	52 ± 2.2<br>*0.200*
**Platelet Count ($x10^3$)**	Mean ± SEM<br>*p-value*	1293 ± 145	1078 ± 208<br>*0.217*	1043 ± 173<br>*0.156*	1098 ± 139<br>*0.185*
**Sodium (mEq/L)**	Mean ± SEM<br>*p-value*	151.2 ± 0.9	150.7 ± 0.25<br>*0.320*	149.2 ± 1.1<br>*0.110*	150.5 ± 1.15<br>*0.320*
**Potassium (mEq/L)**	Mean ± SEM<br>*p-value*	7.5 ± 0.25	7.1 ± 0.45<br>*0.268*	6.9 ± 0.4<br>*0.166*	7 ± 0.25<br>*0.149*

**Chloride (mEq/L)**	Mean ± SEM<br>*p-value*	107.7 ± 0.45	108.5 ± 1.65<br>*0.345*	107 ± 0.55<br>*0.179*	107 ± 0.4<br>*0.140*
**Bicarbonate (mEq/L)**	Mean ± SEM<br>*p-value*	9.5 ± 1.3	7.7 ± 1.4<br>*0.202*	9 ± 2.45<br>*0.433*	9.5 ± 1.9<br>*0.500*
**BUN (mg/dL)**	Mean ± SEM<br>*p-value*	33 ± 2	40 ± 1.5<br>*0.131*	26 ± 1<br>*0.014*	27 ± 0.8<br>*0.021*
**Creatine (mg/dL)**	Mean ± SEM<br>*p-value*	0.2 ± 0	0.2 ± 0<br>*0.146*	0.2 ± 0<br>*0.999*	0.2 ± 0<br>*0.999*
**Glucose (mg/dL)**	Mean ± SEM<br>*p-value*	230.2 ± 16.4	207.5 ± 8.25<br>*0.139*	243.7 ± 23.8<br>*0.330*	211.5 ± 3.45<br>*0.170*
**Calcium (mg/dL)**	Mean ± SEM<br>*p-value*	11.2 ± 0.05	11.1 ± 0.075<br>*0.262*	11 ± 0.18<br>*0.249*	10.9 ± 0.09<br>*0.102*
**Phosphorus (mg/dL)**	Mean ± SEM<br>*p-value*	13.7 ± 0.65	14.5 ± 0.6<br>*0.196*	13.3 ± 0.7<br>*0.379*	12.8 ± 0.4<br>*0.160*
**AST (IU/L)**	Mean ± SEM<br>*p-value*	211 ± 40	*428* ± 102<br>*0.060*	*303* ± 206<br>*0.344*	*121* ± 41<br>*0.083*
**ALT (IU/L)**	Mean ± SEM<br>*p-value*	110 ± 44	114 ± 25<br>*0.472*	124 ± 85.5<br>*0.447*	44 ± 5<br>*0.115*
**Total Bilirubin (mg/dL)**	Mean ± SEM<br>*p-value*	0.1 ± 0	0.1 ± 0<br>*0.999*	0.12 ± 0.025<br>*0.196*	0.1 ± 0<br>*0.999*
**ALP (IU/L)**	Mean ± SEM<br>*p-value*	155 ± 6	126 ± 8<br>*0.065*	138 ± 10<br>*0.115*	140 ± 9.5<br>*0.133*
**Total Protein (g/dL)**	Mean ± SEM<br>*p-value*	5.2 ± 0.1	5.1 ± 0.05<br>*0.320*	4.9 ± 0.05<br>*0.084*	5.2 ± 0.1<br>*0.450*

**Albumin (g/DL)**	Mean ± SEM<br>*p-value*	3 ± 0.05	3 ± 0.05<br>*0.248*	2.8 ± 0.05<br>*0.023*	3 ± 0.05<br>*0.248*
**Globulin (g/dL)**	Mean ± SEM<br>*p-value*	2.1 ± 0.05	2.1 ± 0.05<br>*0.500*	2 ± 0.025<br>*0.122*	2.2 ± 0.05<br>*0.347*
**CPK (U/L)**	Mean ± SEM<br>*p-value*	827 ± 156.5	3582 ± 1698<br>*0.102*	1108 ± 693<br>*0.358*	613 ± 313.5<br>*0.286*

N=4 animals per treatment group. No statistically significant differences were found with rhNELL-1 treatment, although a non-significant trend toward reduced BUN (Blood Urea Nitrogen) was observed. Data is presented as mean ± standard error of the mean. *$P<0.0008$ in comparison to 0 mg/kg/day group.

Supplementary Notes (Supplementary Acronym List):

Ad-Nell-1	Adenoviral Nell-1
ALP	Alkaline Phosphatase
ANOVA	Analysis of Variance
ASBMR	American Society for Bone and Mineral Research
AR	Alizarin Red
AST	Aspartate Aminotransferase
ALT	Alanine Aminotransferase
AVN	Avascular Necrosis
β-TCP	β-Tricalcium Phosphate
BMC	Bone Mineral Content
BMD	Bone Mineral Density
BMP	Bone Morphogenetic Protein
BMSC	Bone Marrow Mesenchymal Stem Cells
B.Ar	Bone Area
B.Pm	Bone Perimeter
BrdU	Bromodeoxyuridine
BUN	Blood Urea Nitrogen
BV	Bone Volume
BV/TV	Bone Volume / Tissue Volume
CPC	Cetylpyridinium Chloride
CPK	Creatine Phosphokinase
Ct.Wi	Cortical Width
Ct.Th	Cortical Thickness
CT	Computed Tomography
CTX	C-Terminal Telopeptide
DKK-1	Dickkopf related protein 1
DXA	Dual Energy X-ray Absorptiometry
EDTA	Ethylenediamine Tetra-acetic Acid
ERK1/2	Extracellular Signal-Related Kinase 1/2
FBS	Fetal Bovine Serum
FEA	Finite Element Analysis
GFP	Green Fluorescent Protein
GMT	Goldner’s Modified Trichrome
H&E	Haemotoxylin and Eosin
HCT	Hematocrit

Hgb	Hemaglobin
HU	Hounsfield Units
IDI	Indentation Distance Increase
JNK	c-Jun N-terminal Kinase
MAPK	Mitogen-Activated Protein Kinase
M-CSF	Macrophage Colony-Stimulating Factor
NMR	Nuclear Magnetic Resonance
OB	Osteoblast
Ob.N	Osteoblast Number
OC	Osteoclast
Oc.N	Osteoclast Number
OCN	Osteocalcin
OPG	Osteoprotegerin
OPN	Osteopontin
OVX	Ovariectomy
PBS	Phosphate Buffered Saline
pCi	Pico Curie
PET	Positron Emission Tomography
PINP	Procollagen I N-terminal Propeptide
PTH	Parathyroid Hormone
qRT-PCR	Quantitative Real Time Polymcrase Chain Reaction
RANKL	Receptor Activator of Nuclear Factor K B Ligand
RBC	Red Blood Cells
rhNELL-1	Recombinant human NELL-1
ROI	Region of Interest
RUNX2	Runt-related Transcription Factor 2
SEM	Scanning Electron Microscopy
SEM	Standard Errors of the Mean
SD	Sprague-Dawley
SOST	Sclerostin
TID	Total Indentation Distance
Tb.Ar	Trabecular bone Area
Tb.N	Trabecular bone Number
Tb.Pm	Trabecular bone Perimeter
Tb.Sp	Trabecular bone Spacing
Tb.Wi	Trabecular bone Width
TRAP	Tartrate Resistant Acid Phosphatase

VKMT	Von Kossa MacNeal's Tetrachrome
VOI	Volume of Interest
WBC	White Blood Cells

Supplementary References:

1. Shen J, *et al.* NELL-1 promotes cell adhesion and differentiation via Integrinbeta1. *Journal of cellular biochemistry* **113**, 3620-3628 (2012).

2. Ieguchi K, *et al.* Direct binding of the EGF-like domain of neuregulin-1 to integrins ({alpha}v{beta}3 and {alpha}6{beta}4) is involved in neuregulin-1/ErbB signaling. *J Biol Chem* **285**, 31388-31398 (2010).
